# Linking Core Promoter Classes to Circadian Transcription

**DOI:** 10.1371/journal.pgen.1006231

**Published:** 2016-08-09

**Authors:** Pål O. Westermark

**Affiliations:** Institute for Theoretical Biology, Charité –Universitätsmedizin Berlin, Berlin, Germany; Center for Integrative Genomics, SWITZERLAND

## Abstract

Circadian rhythms in transcription are generated by rhythmic abundances and DNA binding activities of transcription factors. Propagation of rhythms to transcriptional initiation involves the core promoter, its chromatin state, and the basal transcription machinery. Here, I characterize core promoters and chromatin states of genes transcribed in a circadian manner in mouse liver and in *Drosophila*. It is shown that the core promoter is a critical determinant of circadian mRNA expression in both species. A distinct core promoter class, strong circadian promoters (SCPs), is identified in mouse liver but not *Drosophila*. SCPs are defined by specific core promoter features, and are shown to drive circadian transcriptional activities with both high averages and high amplitudes. Data analysis and mathematical modeling further provided evidence for rhythmic regulation of both polymerase II recruitment and pause release at SCPs. The analysis provides a comprehensive and systematic view of core promoters and their link to circadian mRNA expression in mouse and *Drosophila*, and thus reveals a crucial role for the core promoter in regulated, dynamic transcription.

## Author Summary

Circadian rhythms switch gene expression on and off with a daily rhythm in most tissues in mammals and other animals. Typically, thousands of genes are affected, and the functions of these rhythms include preparing and adjusting various physiological functions in tissues to meet time-of-day dependent requirements optimally. The controllers of the rhythms are often transcription factors (proteins which regulate transcription), which are relatively well known. However, there is a layer between transcription factor action and transcriptional activity whose role in circadian transcription has not previously been characterized: the core promoter. The core promoter acts as a template for the assembly of the intricate machinery that governs initiation of transcription. There are different types of core promoters that are typically used for different types of genes. It is not known which types of core promoters are used for the rhythmically induced genes. Here, it is shown that there are specific characteristics of core promoters driving rhythmic transcription in mice and flies. Furthermore, it is shown that there is a class of strong circadian promoters in mice that drive strong rhythms with very intense average transcriptional rates. These results help understanding the regulatory systems governing circadian transcription, and ultimately, aid the understanding of many diseases resulting from disturbed circadian rhythms.

## Introduction

In many metazoans, transcription of numerous genes in most cell types occurs in a rhythmic fashion with a period of ~24 hours, also if the organism is held under constant conditions; these rhythms are termed circadian transcriptional rhythms [1]. They are to a large extent orchestrated by the cellular circadian clock, which consists of connected feedback loops of clock genes that code for clock proteins. In turn, clock proteins repress or activate transcription of themselves or other clock genes, in this way generating the rhythms [2]. When organisms are held under rhythmic conditions, such as 12hr/12hr light-dark cycles, the circadian clocks in their cells become synchronized to these external so-called zeitgebers. Strictly, one then speaks of diurnal rhythms in gene expression, although here we use the term circadian rhythms also for this case.

The cellular circadian clock becomes manifest in an output of rhythmic mRNA expression of typically hundreds or thousands of clock-controlled genes (CCGs) [3]. The CCGs are thought to be controlled to a large extent by transcription factors (TFs) or coregulators with rhythmic abundances that are part of the cellular circadian clock. In mammals, prominent examples are the CLOCK/BMAL1 heterodimer, which binds to E-boxes in promoters of its target genes [4], the nuclear receptors REV-ERB α and β, which bind to ROR elements [5], and DBP and E4BP4, which bind to D-boxes [6,7]. In the fly *Drosophila melanogaster*, the CLOCK homolog CLK has a similar function, also binding to E-boxes [8]. Rhythmic binding of these core circadian clock TFs (CTFs) and their coregulators to promoters are thought to induce rhythms in transcriptional activities, which ultimately lead to rhythms in mRNA and protein abundances.

Circadian transcriptional rhythms have been investigated by combined analysis of transcript abundances and CTF binding to corresponding promoters. Mouse liver and *Drosophila* have served as useful model systems for investigating transcriptional rhythms, and by for instance comparing nascent transcript to mature mRNA abundances, the propagation of rhythms from transcriptional activities to transcript abundances can be monitored [9–12]. Analysis of such data has revealed a contribution of post-transcriptional regulation to the generation of rhythms in mature mRNA abundances, but transcriptional rhythms remain the dominant determinant of the rhythmic transcriptome [13,14]. Circadian rhythm generation is correlated with rhythmic binding of CTFs to binding sites in the promoters of genes with circadian mRNA expression: for instance, BMAL1 binding phases (peak time), measured at the promoter level in mouse liver, correlate well with phases of the corresponding transcripts [4]. On the bioinformatics and data analysis side, position weight matrix (PWM) based prediction of TF binding sites at the promoter population level paired with quantitative modeling has led to additional insights into the combinatorial regulation of rhythmic transcription by CTFs and also other circadian TFs [15–17]. However, the layer of mechanistic rhythm propagation between CTF binding and transcriptional rhythms has not been characterized systematically. This layer consists of the core promoter and its chromatin environment, the latter which entails specific nucleosome arrangements and histone tail modifications. There are different classes of core promoters, and a question that has not yet been addressed is whether certain types of core promoters are more suitable than others to propagate rhythmic TF binding to rhythmic transcriptional activities.

Core promoters often contain specific binding sites (core promoter elements) for general transcription factors (GTFs) such as TFIID and TFIIB [18,19]. GTFs together with RNA polymerase II (Pol II) nucleate the pre-initiation complex (PIC), which assembles at the transcription start site (TSS) before transcription can initiate. Different GTFs play different roles in PIC nucleation and initiation of transcription [20]. The TFIID subunit TATA-binding protein (TBP) binds to the TATA box, a core promoter element conserved from archaea to mammals, situated ~30 bp upstream of the TSS. The TFIIB subunit binds to BRE elements, located closely upstream (BREu) or downstream (BREd) of the TATA box, and helps recruiting Pol II. Other GTFs help opening DNA at the promoter to form a so-called transcription bubble, and also phosphorylate Pol II at its C-terminal domain and prime it for transcription. Various combinations of core promoter elements are thought to modulate the propagation of TF binding to transcriptional activation, in this manner helping to specify developmentally regulated or tissue specific transcription [21].

Eukaryotic core promoters are often thought to have an inactive ground state, assured by a tendency for nucleosomes to cover promoter DNA. In this ground state, the TSS is assumed to be covered by a nucleosome which constitutes a certain barrier for Pol II to penetrate [22–24]. Transcriptional activators recruit various chromatin remodeling factors, which facilitate PIC assembly and transcriptional initiation by loosening the nucleosomal barrier. This can happen through e.g. histone acetylation and ATP-dependent nucleosomal displacement, as shown for the human IFN-β promoter [25]. One mode of circadian regulation of transcription could then be rhythmic binding and unbinding of transcription factors, resulting in rhythmic recruitment of histone modifiers and nucleosome remodelers that in turn free up the TSS for PIC formation and transcription initiation in a rhythmic fashion. Such a mode is indeed consistent with several hallmarks of the strongly circadian *Dbp* transcript, including observed rhythmic CLOCK/BMAL1 binding, histone H3 Lys4 trimethylation (H3K4me3) and Lys9 acetylation modifications, as well as rhythmic gross H3 abundance at the TSS [26]. However, a subset of metazoan promoters appear to have an active ground state, in the sense that they are depleted of nucleosomes and bound by Pol II even when there is no active transcription. A signature of such promoters is a high level of Pol II immediately downstream of the TSS (as detected by ChIP-Seq), compared to the Pol II level in the gene body [27,28]. The TSS to gene body Pol II ratio is referred to as pausing index, since Pol II has often already engaged in initial transcription at these promoters but sits paused ~50 bp downstream of the TSS. Paused Pol II is specifically detectable by techniques such as global run-on sequencing (GRO-Seq) as sharp peaks of nascent mRNA fragments aligning just downstream of the TSS [29]. There are regulated processes promoting such pausing, which involve pausing factors such as DSIF and NELF. Induction of transcription at these TSSs involves pause release factors such as P-TEFb [30–32].

There are several correlated hallmarks of promoters with active ground state, including nucleosome-depleted regions immediately upstream of the TSSs, absence of TATA box, high levels of Pol II and the H3K4me3 mark immediately downstream of the TSS, as well as high levels of the H2A.Z histone variant at the first nucleosome downstream of the TSS [28,33–39]. In mammalian promoters with active ground state, there is the additionally associated hallmark of high observed to expected ratios of CpG dinucleotides around the TSS (hereafter: CpG ratios). The distribution of this ratio computed for promoters is bimodal, motivating a classification of promoters as either having low or high CpG ratios [40,41]. The causal relationships between these hallmarks are debated and not easily teased apart. Probably, causality is cyclical in some cases: for instance, Pol II recruits the H3K4 methylases SET1 and MLL1 at some promoters [42], while H3K4me3 in turn is able to actively recruit the TAF3 subunit of TFIID, a part of the Pol II pre-initiation complex [43]. There are also several other mechanisms that help explain the correlated hallmarks of promoters with active ground state. Non-methylated CpG dinucleotides may recruit the protein CFP1, which in turn is associated with SET1, resulting in increased H3K4me3 levels [44,45]. The H3K4me3 mark may help recruit ATP-dependent chromatin remodelers to influence nucleosome positioning [46]. Certain TF coregulators, nucleosome-depleted regions, and ATP-dependent chromatin remodelers may elevate levels of the H2A.Z variant at the +1 nucleosome [47,48]. Although a debated issue, *in vitro* and *in vivo* evidence collected to date suggest CpG dinucleotides help instruct the formation of nucleosome-depleted regions of mammalian promoters [49–51]. Besides this DNA sequence feature, proteins associated with CpG-rich DNA as well as ATP-dependent chromatin remodelers probably help to establish nucleosome-depleted regions in vivo [46,50,52]. In particular, TSS-bound Pol II, perhaps in a paused state, could contribute to establishing nucleosome-depleted regions by sterically hindering nucleosome formation in a competitive fashion. This effect is probably more pronounced at nucleosome-depleted regions at CpG-poor promoters, with their higher propensity for nucleosome coverage [50,53].

Based on these myriad features and mechanisms, two main metazoan promoter classes, type I and type II promoters, have been proposed [54]. Type I promoters drive regulated, tissue-specific mRNA expression, have an inactive ground state with a less pronounced nucleosome-depleted region, are enriched for TATA boxes, and are depleted of CpG dinucleotides in vertebrates [55,56]. These promoters are often focused, which means that they have a well defined TSS, as measured with the CAGE assay [57]. Type II promoters drive ubiquitously expressed transcripts, have more pronounced nucleosome-depleted regions, are enriched for the H3K4me3 mark, CpG dinucleotides, and are depleted of TATA boxes. These promoters are often dispersed, meaning that they have multiple TSSs scattered over regions of tens or hundreds of bp [55,56]. They are also enriched for constitutively transcribed ("housekeeping") genes [41], presumably because their nucleosome-depleted regions are conducive to constitutive transcription.

Both type I and type II promoters may govern inducible transcription, however implemented in different manners, as suggested by two studies of LPS-induced transcription of ~50 genes in mouse macrophages [58,59]. Primary response genes, which are quickly induced without requirement for additional protein synthesis, tend to have type II promoters, and induction tends to occur by induced release of paused Pol II. Secondary response genes, which are more slowly induced with requirement for additional protein synthesis, tend to have type I promoters, require ATP-dependent chromatin remodeling, and to have an inactive basal state without bound Pol II with a nucleosome blocking PIC assembly. Open questions include whether circadian transcription–a particular mode of inducible expression–preferably employs type I or type II promoters, to which degree these rhythms are accompanied by oscillations on the associated chromatin hallmarks at the core promoters, and which mechanisms might be involved in circadian regulation of transcription at the core promoter.

**Fig 1:**
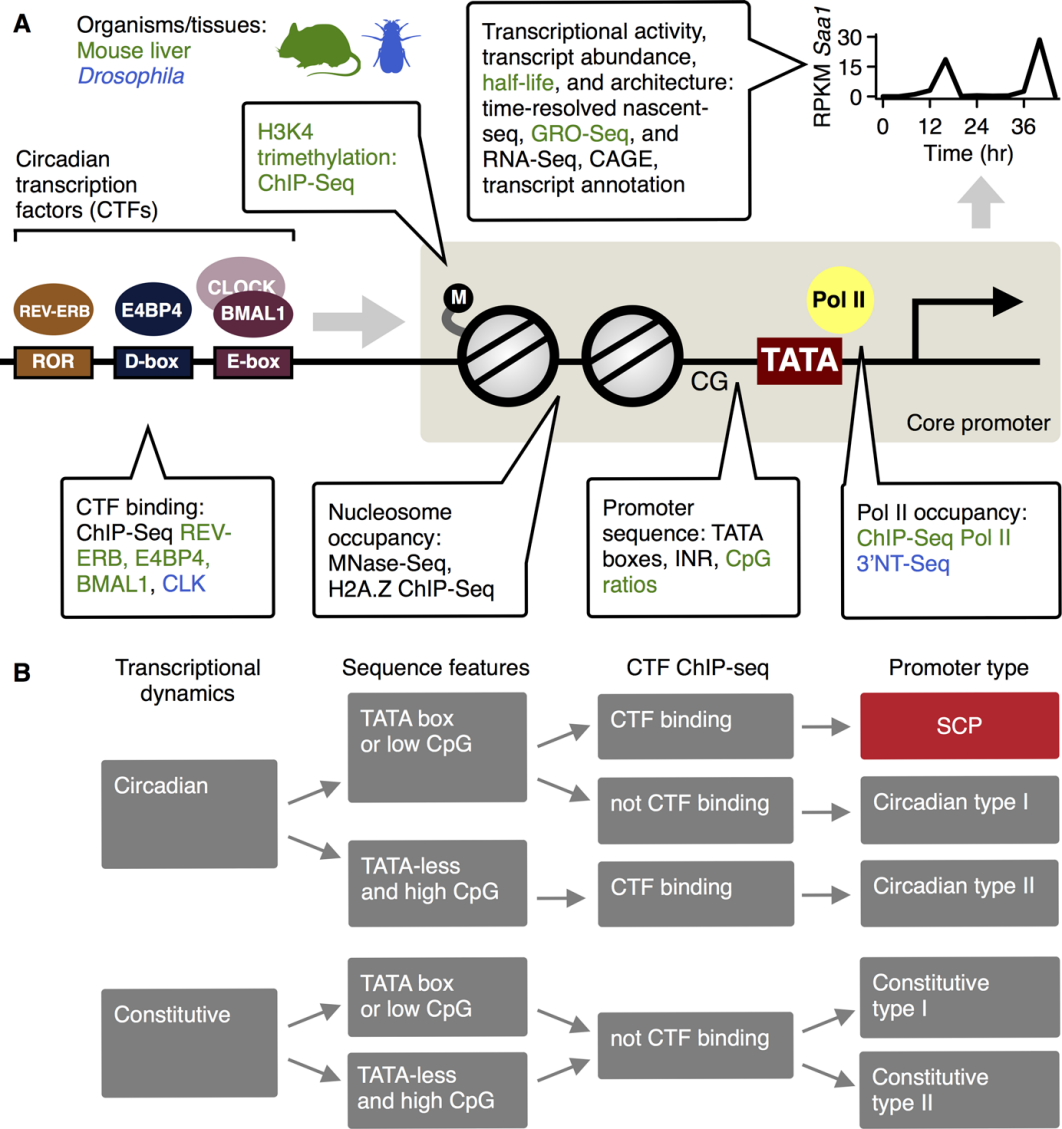
**A. Overview of the study and the various data sources (listed in the callouts).** Data were compiled from published studies of mouse liver in animals held under rhythmic light-dark (LD) conditions (green), or of *Drosophila* (blue), or both (black). Rhythmic transcriptional activities were estimated from Nascent-Seq data as visualized for the *Saa1* transcript. Rhythmic transcriptional activities thought to be generated by circadian transcription factors (CTFs) such as REV-ERB α and β, E4BP4, and the CLOCK/BMAL1 heterodimer in mouse, or CLK in *Drosophila*. The core promoter (beige box) mediates rhythms in CTF binding to rhythmic transcription initiation. This mediation may involve chromatin and nucleosomal remodeling, interacting with rhythms in general transcription factor and Pol II binding to core promoter elements such as the TATA box. B. Overview of promoter classification. Promoters were classified according to whether they drive circadian or constitutive transcription, according to core promoter sequence features, and according to detected CTF binding at the promoter.

This report describes the core promoters of genes transcribed in a circadian manner (hereafter: circadian promoters) in mouse liver and in *Drosophila*, by integrating promoter sequence features (CpG ratios and core promoter elements) with a wealth of genome-wide data measuring chromatin state, transcriptional activities, and CTF binding (Fig 1A). It is shown that the core promoter is of greatest importance for circadian transcription. In particular, a core promoter class in mouse combining hallmarks of type I and type II promoters is uncovered. This promoter class, here termed strong circadian promoter, drives circadian transcription with both high amplitudes and high average transcriptional rates.

## Results

### Core promoters of circadian genes are CpG-depleted and strongly enriched for TATA boxes

To investigate whether there is a preferred core promoter architecture for circadian promoters in mammals, a collection of mouse transcripts assignable to unique TSSs and promoters was established (RefSeq transcript annotation, Methods). The transcriptional activities producing these transcripts were estimated by reanalyzing Nascent-Seq data [11]. These data were collected from mouse livers sampled every 4 hrs under 12 hr/12 hr light-dark (LD) conditions (time points hereafter referred to as zeitgeber time, ZT). To avoid confounding effects from lighting conditions, only data obtained from mice held under LD were used in the present study. A group of 1895 promoters with very clear rhythmic transcriptional activities (Methods) were classified as circadian promoters. In contrast, a background group of 5829 promoters with significant transcriptional activities yet without any signs of circadian rhythms were classified as constitutive promoters. A group of 4892 silent promoters without detectable or with very low transcriptional activities was also identified. For rhythmic activities, relative amplitudes (absolute amplitudes divided by means, hereafter: amplitudes) were estimated by harmonic regression (Methods) and used for the further analysis.

To characterize core promoters, areas in the vicinities of the TSSs were scanned for the presence of TATA boxes, BREu, and BREd motifs (Methods and S1A Fig). In addition, CpG ratios were quantified (Methods). Furthermore, MNase-Seq data [60], which quantify nucleosome occupancies, were reanalyzed. A summary statistic for nucleosome occupancy for each promoter was formed by averaging nucleosome coverages between −101 and −1 bp from the TSS. Finally, mouse liver CAGE data, which measure precise TSS usage by transcripts [57], were used to classify promoters as focused or dispersed (Methods). Focused promoters have a well-defined TSS: Most transcripts start within a narrow range of a few bp. Dispersed promoters give rise to transcripts with starting positions varying by tens of bp. These three characteristics–core promoter sequence features and motifs, nucleosome occupancy, and TSS variation–have previously been used to characterize different promoter classes, and were used as a starting point for the present study.

**Fig 2:**
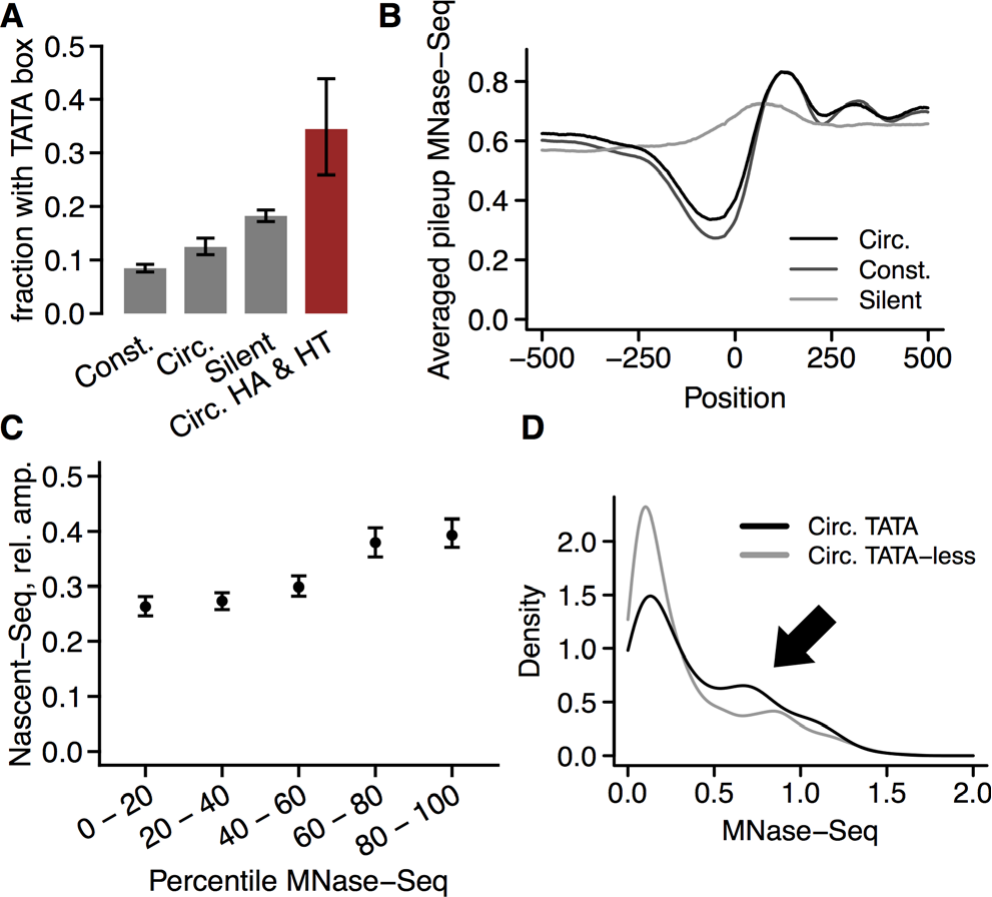
Basic properties of circadian promoters. A. Fractions promoters with TATA box for different promoter classes, with 95% confidence intervals assuming binomial distributions. Legend: const. = constitutive; circ. = circadian; circ. HA & HT = circadian with high amplitude and high average transcriptional activity (upper quartiles, Methods). Bars from left to right: *n* = 5829, 1895, 4892, 116. B. Averaged nucleosome occupancies for different promoter classes. Legend as in panel A. Position 0 refers to the TSS. Pileup refers to normalized MNase-Seq reads aligned to a given position. Median pileups for each position and promoter class are visualized. C. Transcriptional amplitude increases with nucleosome occupancy. Mean pileups for all circadian promoters at positions 101 to 1 bp upstream of the TSS were ranked and divided into 5 quantiles. Amplitudes in each such quantile are visualized (medians with 95% confidence intervals). D. Distribution of nucleosome occupancies immediately upstream of the TSSs of circadian promoters. Especially circadian promoters with TATA box appear to have a low occupancy subpopulation coexisting with a subpopulation with high nucleosome occupancies (arrow).

Circadian promoters turned out to be enriched for TATA boxes when compared to constitutive promoters (Fig 2A, Fisher's exact test, p < 5×10^−7^, odds ratio 1.54), although not as strongly enriched as silent promoters. However, circadian promoters driving transcription with high amplitudes and high average activity were exceptionally strongly enriched for TATA boxes, with around 35% TATA box containing promoters (Fig 2A). Circadian promoters had a slightly less pronounced nucleosome-depleted region immediately upstream of the TSS than constitutive promoters (rank sum test, p < 10^−15^, median ratio 1.24), but were significantly more nucleosome-depleted than silent promoters (Fig 2B). There was a positive correlation between nucleosome occupancy immediately upstream of the TSS (hereafter: nucleosome occupancy) and transcriptional amplitude (Spearman's rho = 0.28, p < 10^−15^, Fig 2C). CpG ratios were negatively correlated with transcriptional amplitudes (S1B and S1C Fig). Circadian promoters had overall slightly lower CpG ratios than constitutive promoters (rank sum test, p < 10^−13^, median ratio 0.93), but silent promoters had much lower CpG ratios than circadian promoters (S1D Fig). In agreement with these correlations and with previous studies [51,59], nucleosome occupancy was negatively correlated with CpG ratio (Spearman's rho = −0.45, p < 10^−15^ S1E Fig).

Consistent with their enrichment for TATA boxes, circadian promoters also tended to be more focused than constitutive promoters according to the CAGE data (Fisher's exact test, p < 0.002, odds ratio 1.24). Circadian TATA box promoters were furthermore depleted of the BREd motif compared to constitutive TATA box promoters (Fisher's exact test, p = 0.02, odds ratio 0.68), or when comparing circadian to constitutive promoters overall (Fisher's exact test, p < 0.0033, odds ratio 0.85). No enrichment or depletion was detected for the BREu motif in circadian promoters compared with constitutive promoters (Fisher's exact test, p = 0.27, odds ratio 0.93). Thus, the BREd motif appears to favor constitutive mRNA expression.

Taken together, these findings seem to indicate that circadian promoters, especially those that drive transcriptional rhythms with high amplitude, tend to be of the type I class. However, the distribution of nucleosome occupancies for circadian promoters with TATA box was broad and bimodal (Fig 2D). Clearly, a large population of circadian promoters with TATA box had a nucleosome-depleted region upstream of the TSS, a hallmark thought to be more typical of type II promoters, while a group of other circadian promoters had much higher nucleosome occupancies (Fig 2D, arrow). As is the case in human cells [51,61], there was a general negative correlation between nucleosome occupancy and transcriptional activity among all promoters corresponding to expressed transcripts (Spearman's rho = −0.34, p < 10^−15^, S1F Fig). Consistent with this and the positive correlation between amplitude and nucleosome occupancy, there was among circadian promoters also a negative correlation between mean transcriptional activity and amplitude (Spearman's rho = −0.28, p < 10^−15^). However, although circadian promoters had higher nucleosome occupancy on average compared to constitutive promoters (Fig 2B), they did not have lower median transcriptional activity (rank sum test, p = 0.13, median ratio 1.03). Thus, circadian promoters might harbor separate promoter classes that are not detectable by averaging measurements over the entire promoter group. In particular, there might be a hidden group of circadian promoters with atypically high transcriptional activities, increasing the population average transcriptional activity of circadian promoters.

### Identifying a separate class of strong circadian core promoters

To stratify circadian promoters further and uncover possible hidden groups, a reanalysis was performed of mouse liver ChIP-Seq data measuring the genome-wide binding of core circadian clock transcription factors (CTFs) known to potently induce rhythmicity in transcription of CCGs. CTFs considered were REV-ERB α and REV-ERB β (which bind to ROR elements), E4BP4 (which binds to D-boxes) and BMAL1 (which binds to E-boxes), and promoters were scanned ± 3000 bp of the TSS for CTF binding events (Methods). The reason for this stratification was that CTFs levels exhibit strong circadian rhythms in most tissues. Promoters not binding CTFs but still driving circadian transcriptional rhythms in mouse liver are probably controlled by other rhythmic TFs or cofactors that could be induced partly by rhythmic external cues in an inducible, tissue-specific manner [16]. Thus, core promoter architecture could differ between CTF and non-CTF binding promoters. Finally, based on their bimodal CpG ratio distribution, promoters were classified as high CpG ratio (HCpG) or low CpG ratio (LCpG) promoters (Fig 2D and Methods).

**Fig 3:**
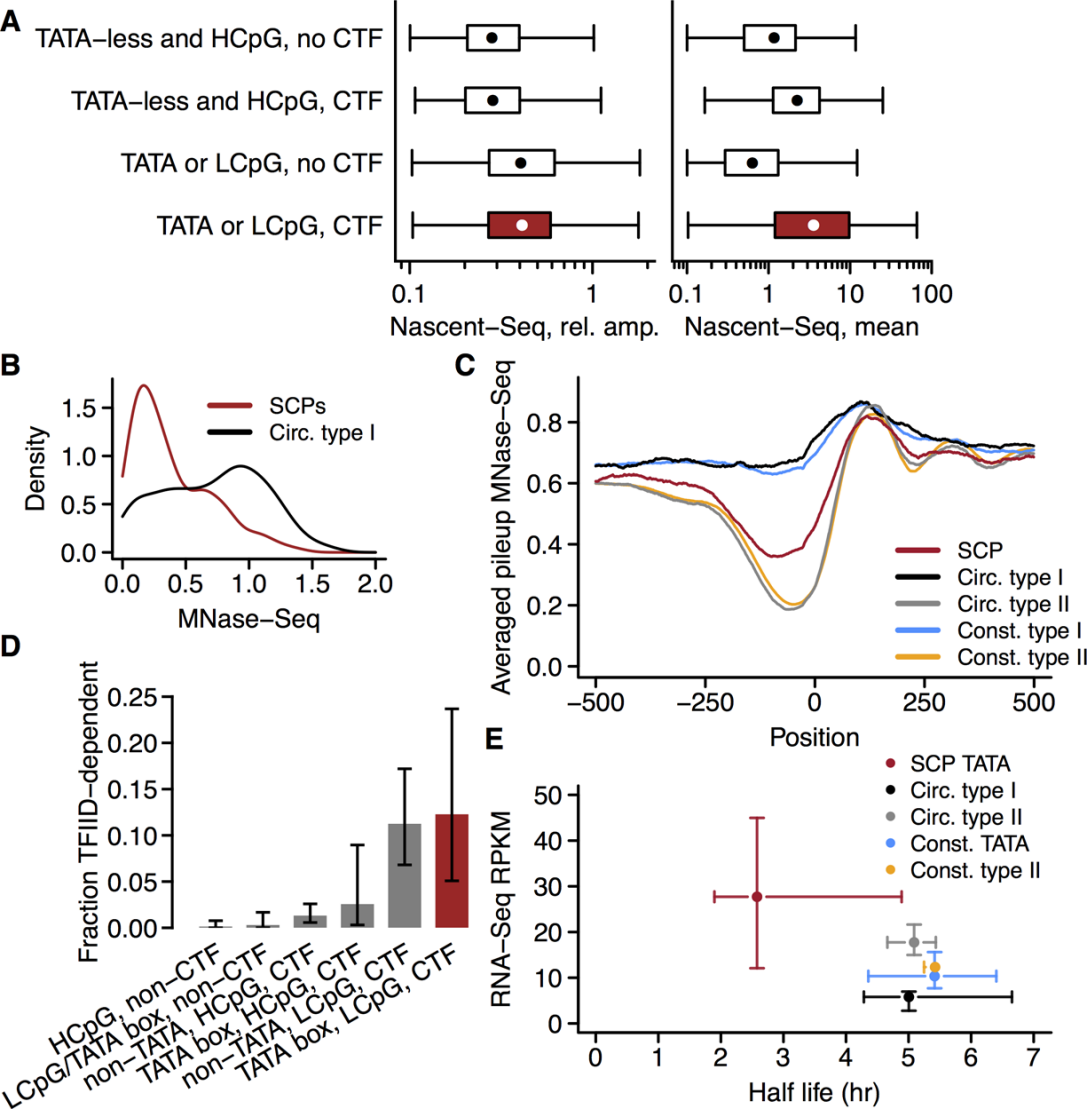
Identifying strong circadian promoters (SCPs). A. Amplitudes and averages of transcriptional activities as measured by Nascent-Seq were quantified for the transcript corresponding to each promoter. Promoters were stratified as either TATA box-containing and/or LCpG, or TATA-less and HCpG, as well as exhibiting either no, or at least one CTF ChIP-Seq peaks (CTFs, circadian transcription factors BMAL1, REV-ERBα, REV-ERBβ, and E4BP4). TATA boxes and LCpG were strongly associated with high circadian transcriptional amplitudes, while CTFs rather were associated with high average transcriptional activities. Box plots show medians, 25/75% quantiles, and minimum/maximum values. Promoter groups from top to bottom: *n* = 665, 605, 330, 295. B. Nucleosome occupancies 101 to 1 bp upstream of the TSSs for SCPs and type I circadian promoters (with TATA box or LCpG but not CTF binding). Occupancies were computed from MNase-Seq data (Methods). Data are presented as kernel densities. C. Nucleosome occupancies around TSSs for different promoter classes. Pileups were computed from MNase-Seq data (Methods) and averaged over the promoter classes for each position relative to the TSS (excluding top and bottom 1% values, respectively, due to a few outlier promoters). Circ. = Circadian; Const. = Constitutive; Type I defined as in panel B, type II = CTF binding TATA-less and with HCpG. D. Fraction of TFIID-dependent promoters for different classes of circadian promoters, with 95% confidence intervals assuming binomial distributions. Bars from left to right: *n* = 721, 330, 605, 78, 160, 57. E. Mature mRNA expression levels and transcript half lives for different promoter classes. Mature mRNA expression levels were quantified from poly(A)^+^ RNA-Seq data for the transcript corresponding to each promoter; transcript half lives were compiled from two literature sources (Methods). Mean expression levels and half lives were median averaged over the promoter classes and are shown as points. Error bars represent 95% confidence intervals for the medians. Circ. = Circadian; Const. = Constitutive; TATA = with TATA box; type I and type II as in panels B and C.

As expected, circadian promoters were enriched for CTFs compared to constitutive promoters (Fisher's exact test, p < 10^−10^, odds ratio 1.44). Further, CTF-binding circadian promoters (n = 900) were enriched for TATA boxes, compared to circadian promoters not binding CTFs (n = 995, Fisher's exact test, p = 0.0017, odds ratio 1.56). Unexpectedly, however, CTF binding alone was not associated with high transcriptional amplitude (Fig 3A, left, rank sum test, p = 0.57, median ratio 0.99). Rather, TATA boxes and LCpG were associated with high transcriptional amplitudes, regardless of CTF binding (rank sum test, p < 10^−15^, median ratio 1.4). In contrast, CTF binding was strongly associated with high average transcriptional activities (Fig 3A, right, rank sum test, p < 10^−15^, median ratio 2.4), independently of TATA boxes or LCpG. These effects were additive: Promoters with both CTF binding and a TATA box or LCpG were associated with very strong transcription, with high amplitudes as well. Note that the scale is logarithmic and that the fold differences in mean transcriptional activities are quite large. The same effects were observed when stratifying for TATA boxes or CpG content separately (S2A Fig).

CTF-binding circadian promoters with either LCpG or TATA box are hereafter referred to as strong circadian promoters (SCPs, listed in S1 Table), in light of the marked association of these sequence features with both strongly rhythmic and high transcriptional activities.

### SCPs have distinct, atypical combinations of core promoter features

To investigate and characterize SCPs further, a search for distinguishing characteristics of these promoters in terms of nucleosome occupancy and sequence features was carried out. By stratifying all circadian promoters with LCpG or TATA box into either SCPs or non-CTF binding promoters (Fig 1B), the broad bimodal nucleosome occupancy distribution (Fig 2F) was resolved into the population of SCPs, which had low nucleosome occupancy, and the population of non-CTF binding LCpG or TATA box containing circadian promoters, which had high nucleosome occupancy (Fig 3B). In stark contrast to the general anti-correlation between promoter nucleosome occupancy and CpG ratio, SCPs turned out combine low average CpG ratios with low nucleosome occupancies (S2B Fig). SCPs had, in fact, almost as low nucleosome occupancy as constitutive TATA-less HCpG promoters without CTF peaks (hereafter: constitutive type II; Figs 1B and 3C). Thus, SCPs appeared to escape both the negative correlation between amplitude and mean transcriptional activity, as well as between amplitude and nucleosome depletion (Fig 2C). The group of circadian promoters that do not bind CTFs, but with LCpG or TATA box, on the other hand, had characteristics of canonical type I promoters [54]: low median transcriptional activities (Fig 3A) and high nucleosome coverage (Fig 3C). Promoters in this group are hereafter referred to as circadian type I promoters (Fig 1B).

Circadian CTF binding HCpG promoters without TATA box, on the other hand, had the low nucleosome occupancies typical of type II promoters (Fig 3C), which given the initial analysis here were consistent with their low oscillation amplitudes (Fig 3A). These promoters are hereafter called circadian type II promoters (Fig 1B).

For comparison to earlier results on correlations between core promoter properties and nucleosome occupancies [55,56], constitutive promoters with LCpG or TATA box (but without CTF peaks, to exclude residual circadian promoters whose transcriptional rhythms might not have been detected), were also investigated. These promoters (hereafter: constitutive type I promoters, Fig 1B) had the expected high nucleosome occupancies upstream of the TSS, whereas constitutive type II promoters tended to have the familiar pronounced nucleosome-depleted region upstream of the TSS (Fig 3C).

One possibility would be that SCPs simply constitute regular type I promoters that happen to be very strongly transcribed. To exclude this, constitutive type I promoters were sampled (with replacement) to match the distribution of average nascent mRNA read counts of SCPs. This resulted in an artificial group of constitutive TATA box promoters with the same statistical distribution of transcriptional activities as SCPs. A bootstrapping test procedure was then employed to probe whether nucleosome occupancies of the matched constitutive type I promoter population are similar or different to those of SCPs (Methods). This analysis (S2 Table) showed that SCPs indeed have significantly lower nucleosome occupancy than constitutive type I promoters with matched mean transcriptional levels.

Could the low nucleosome occupancy of SCPs be due to BMAL1 binding, since this CTF is known to act as a pioneering factor and induce eviction of nucleosomes [60]? A reanalysis of MNase-Seq data from mouse livers of Bmal1^−/−^ animals [60] yielded nucleosome profiles very similar to those of livers from wild type mice (S2C Fig). This points to other reasons than BMAL1 for the nucleosome depletion immediately upstream to the TSS of SCPs.

Does daily up- and downregulation of transcription require mechanisms associated with focused transcription initiation? As mentioned, regulated and CpG-poor TATA box containing promoters are often focused. However, promoters with an ordered nucleosome pattern and a pronounced nucleosome-depleted region upstream of the TSS are generally more dispersed [54]. This relationship between nucleosome coverage and TSS width could be reproduced with the present data set: dispersed promoters had significantly lower nucleosome occupancy (rank sum test, p < 10^−15^, median ratio 0.81). Thus, since SCPs are CpG-poor and enriched for TATA boxes, but on the other hand have low nucleosome occupancies, it is not obvious if SCPs should tend to be focused or dispersed: the general rules are in conflict. An analysis showed that SCPs with TATA box were mainly focused with a smaller dispersed subpopulation, but that TATA-less SCPs (consequently with LCpG) tended to be much more dispersed (S2D Fig). Thus, strong circadian transcriptional rhythms appear compatible both with focused and dispersed transcriptional initiation.

Since differentiated cells often do not employ TFIID for the majority of transcripts, it is of interest to assess whether SCPs, circadian promoters, or even expressed transcripts in general are associated with this PIC subunit in the adult mouse liver. To do this, the lists of expressed promoters, circadian promoters, and SCPs were cross-referenced with a list of transcripts that are down-regulated in mice with a liver-specific deletion of the TAF10 subunit of TFIID, without which TFIID dissociates [62].

There was a remarkable enrichment for such TFIID-dependent transcripts among transcripts with promoters of expressed transcripts binding CTFs, especially CTF-binding promoters with TATA box. In fact, around 89% of all 130 TFIID-dependent expressed transcripts had CTF binding promoters (S2E Fig), compared to 41% for non-TFIID-dependent expressed transcripts (neither up-regulated nor down-regulated upon TAF10 depletion, Fisher's exact test, p < 10^−15^, odds ratio 12.2). Conversely, focusing on circadian promoters, CTF binding in conjunction with LCpG was associated with TFIID-dependence. 12% of LCpG SCPs were strongly TFIID-dependent, compared to 0.03% for circadian promoters with neither CTF binding nor TATA box or LCpG (Fig 3D). A possible caveat might be that the observed down-regulation thought to be due to TFIID dissociation in fact was due to circadian transcription: samples were not necessarily collected at the same times of day. However, then an overrepresentation of transcripts up-regulated upon TAF10 depletion would also be expected among CTF binding promoters if all phases are roughly equally probable. Such an overrepresentation was not found among the 119 expressed transcripts up-regulated upon TAF10 depletion (48% vs. 41%, Fisher's exact test, p = 0.11, odds ratio 1.35). This suggests that the down-regulation likely indeed is due to TFIID dissociation. These results suggest that CTF binding promoters, in particular those with LCpG, tend to employ TFIID also in fully differentiated hepatocytes to a higher degree than other promoters.

The SCPs appeared to drive extraordinarily high transcriptional activities and amplitudes (Fig 3A). Is this reflected in mature transcript abundances as well? By analyzing mouse liver mature mRNA abundances from RNA-Seq measurements over 2 circadian cycles [11], matching these to the Nascent-Seq transcripts, and estimating mean mRNA expression levels and oscillation amplitudes (Methods), this question could be answered. As shown in Fig 3E, the extraordinarily high mean expression levels and amplitudes of SCPs were indeed carried over to the mature transcript levels. These results were confirmed using an alternative microarray-based data set to quantify mature mRNA abundances [63] (Methods and S2F Fig).

Short mean half lives are an absolute requirement for high circadian rhythm amplitudes in any molecular species [13]. Short half lives tend to decrease mean abundances, which could be counteracted by high transcriptional activities in the case of mRNAs. Cross-referencing the data on mRNA amplitudes with data on mRNA half lives [64,65] showed that mRNAs driven by SCPs with TATA box have extraordinarily short half lives compared to any other class of mRNAs, including constitutively expressed mRNAs driven by either TATA box or TATA-less promoters (Fig 3E). The high mRNA abundances associated with TATA box SCPs are thus reached in spite of short transcript half lives, presumably through their exceptionally high transcriptional activities.

In summary, the results suggest that SCPs might constitute a separate core promoter class. SCPs are characterized by being much more strongly transcribed and by having much lower nucleosome occupancies than standard type I promoters, yet they retain low CpG ratios.

### Exceptionally high and rhythmic paused Pol II levels, but constant and low nucleosome occupancies at SCPs

What may cause the nucleosome depletion upstream of the TSSs of SCPs? It might be the case that promoter-proximal paused Pol II, or associated factors, hinder nucleosome assembly at these promoters [53]. To follow up on this hypothesis here, ChIP-Seq data on Pol II in mouse liver sampled at 7 time points over 24 hours [10] were reanalyzed. Sequencing reads were compiled both for the promoter-proximal pausing region (20–100 bp downstream of the TSS) as well as for the gene body (300–1300 bp downstream of the TSS), which yielded normalized RPKM values for these regions, reflecting the amount of bound Pol II, or Pol II coverage (Methods). This also allowed calculating the pausing index (PI), which is the Pol II coverage in the promoter-proximal pausing region normalized by the gene body Pol II coverage. The PI reflects the tendency of Pol II to remain associated with the TSS for a given level of transcriptional activity. To verify that this is accompanied by truly paused Pol II, the analysis was complemented with GRO-Seq data sampled at 8 time points over 24 hours in duplicates [6], in order to detect peaks of short transcripts produced by paused Pol II.

**Fig 4:**
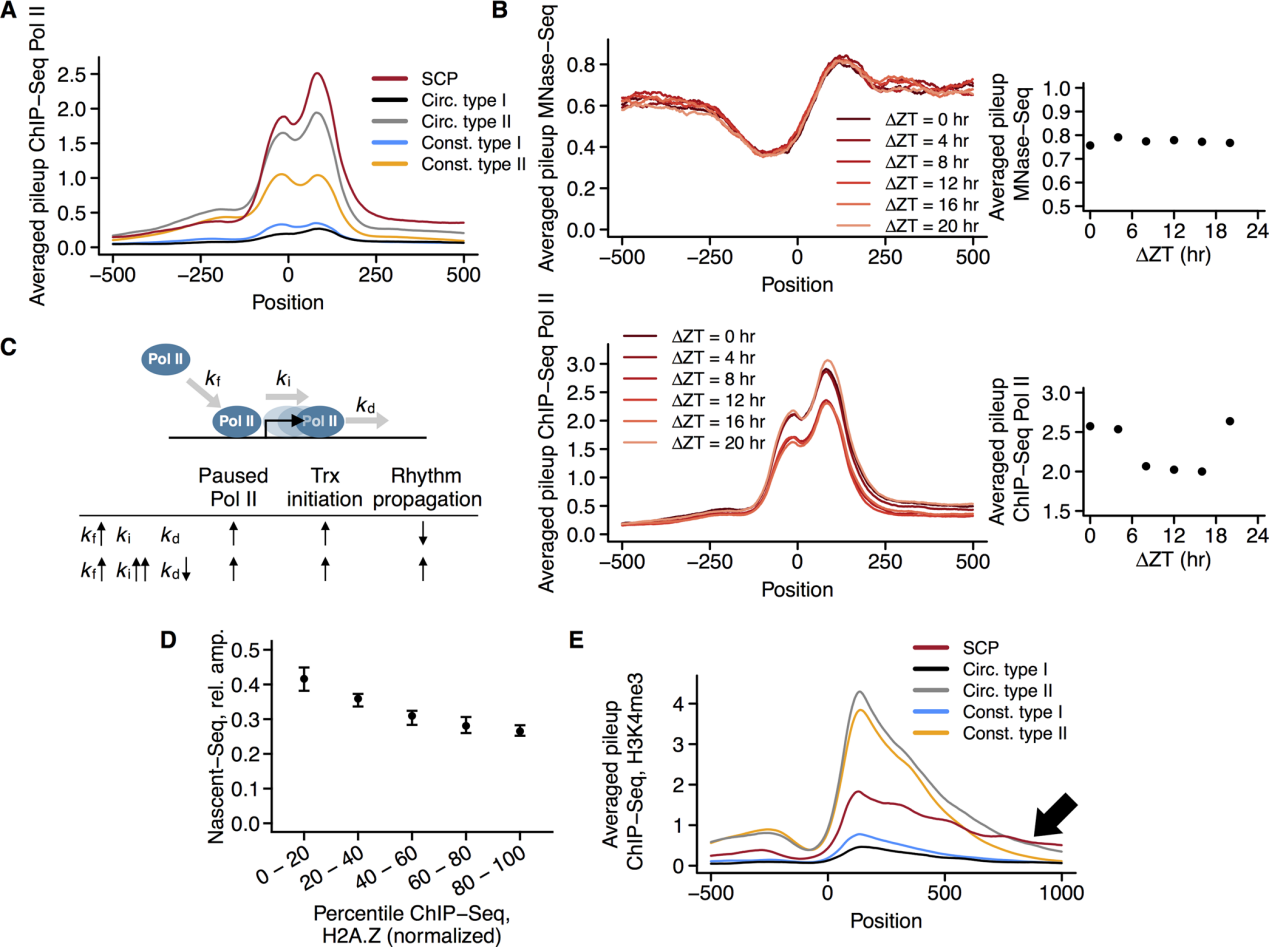
Pol II, nucleosome, and H3K4me3 dynamics at circadian promoters. A. Pol II levels around TSSs for different promoter classes. Pileups were computed from ChIP-Seq data (Methods) and averaged over the promoter classes for each position relative to the TSS. Circ. = Circadian; Const. = Constitutive; NC = non-TATA box, CTF binding; NN = non-TATA box, non-CTF binding. B. Time-resolved pileups of nucleosome occupancies (MNase-Seq data, methods), and Pol II levels (Pol II ChIP-Seq data, Methods) around the TSSs, averaged over all SCP promoters, phase-shifted according to transcriptional phase. Dot plots represents averages over positions 41 to 121 bp downstream of the TSS (nucleosomes), and positions 51 to 151 bp downstream of the TSS (Pol II). The Pol II levels depicted in the dot plot were rhythmic (harmonic regression p = 0.01), but the nucleosome occupancies were not (harmonic regression p = 0.52). C. Summary of the kinetic model analyzed. The model includes recruitment, initiation, and pausing release steps with accompanying rate constants. Increased Pol II recruitment rate only leads to both increased paused Pol II levels and increased rhythm propagation, if combined with increased Pol II initiation rates and decreased Pol II pause release rate constants. D. Transcriptional amplitudes decrease with higher H2A.Z levels. Mean H2A.Z pileups for all circadian promoters at the +1 nucleosome normalized to the +1 nucleosome peak (Methods) were ranked and divided into 5 quantiles for visualization. Medians and 95% confidence intervals of amplitudes in each such quantile are shown. E. H4K3me3 levels around TSSs for different promoter classes. Pileups were computed from ChIP-Seq data (Methods) and averaged over the promoter classes for each position relative to the TSS (excluding top and bottom 1% values, respectively, due to a few outlier promoters). Circ. = circadian; Const. = constitutive. NC = non-TATA box, CTF binding; NN = non-TATA box, non-CTF binding. Arrow highlights the striking high H3K4me3 signal continuing into the gene body.

Indeed, SCPs had higher promoter-proximal Pol II coverage than any other promoter group, including circadian or constitutive type II promoters, even though the latter two promoter groups had slightly lower nucleosome occupancies as SCPs (Fig 4A). These high Pol II levels at SCPs were accompanied by an even stronger peak of GRO-Seq reads (S3A Fig), suggesting that Pol II sits paused but engaged in transcription at these promoters. The bootstrapping test procedure for comparing SCPs to transcriptional activity-matched constitutive type I promoters (above and Methods) showed that this high Pol II coverage is indeed much more common among SCPs as compared to highly expressed type I promoters (p < 10^−6^).

Although there also was a general negative correlation between nucleosome occupancy and paused Pol II, SCPs consistently displayed atypically high levels of paused Pol II for a given nucleosome occupancy (S3B Fig), but not atypically high PIs (S3C Fig). The lower PIs may be attributed to SCPs being associated with higher Pol II gene body levels when compared to other circadian promoters (rank sum test, p < 10^−15^, median ratio 2.3, Fig 4A). This is consistent with the idea that especially SCPs might employ Pol II to maintain a low nucleosome occupancy. It also suggests that Pol II sits mainly in a paused state at SCPs, ready to initiate transcription at appropriate time windows.

One could thus ask whether there are widespread oscillations in the competition between a nucleosome-occupied state and a Pol II-bound nucleosome-depleted state at circadian promoters, and at SCPs in particular. If so, rhythms in nucleosome densities and Pol II levels should be observable in MNase-Seq and Pol II ChIP-Seq data. Since both the Pol II ChIP-Seq data [10] and the MNase-Seq data [60] analyzed here were sampled over the course of one day at 7 and 6 time points, respectively, these data allow a glimpse into the dynamics of nucleosome occupancies and Pol II levels. The sequencing depth of the MNase-Seq data is too low to allow reliable quantification at the single-promoter level; they are, however, readily analyzed at the promoter population level. For this, promoters were binned according to their transcriptional phase as determined by analysis of the Nascent-Seq data (Methods), plus/minus 2 hrs. The MNase-Seq data were reordered temporally so that for each promoter, time point 0 corresponded to the transcriptional phase of the promoter's bin. The same exercise was performed with the Pol II ChIP-Seq data, in order to compare the results properly. Visualizing the resulting 6 phase bins revealed a distinctly static nucleosome landscape around the TSSs of SCPs (Fig 4B, p = 0.52, harmonic regression test for rhythmicity). On the other hand, Pol II levels, analyzed in the same manner, revealed a clear rhythmicity that coincided with the transcriptional phase (Fig 4B, p = 0.01, harmonic regression test for rhythmicity). The lack of nucleosome occupancy rhythms was pervasive also at circadian promoters other than SCPs, and the promoter-proximal Pol II levels had weaker rhythms at these promoters (S3D Fig). Hence, the present analysis showed no evidence that nucleosomal dislocation is commonly employed for generating transcriptional circadian rhythms. However, at SCPs, rhythmic promoter-proximal Pol II levels may reflect an involvement in rhythmic transcriptional regulation. These Pol II rhythms had a very high base line, which might serve to keep these promoters permanently nucleosome-depleted, enabling a high average rate of transcriptional initiation.

How may the strong tendency for promoter-proximal Pol II accumulation be achieved and combined with generation of transcriptional rhythms? To investigate this from a biochemical kinetics perspective, a simple but general mathematical model formulated previously [10] was considered. The model describes Pol II recruitment, transcriptional initiation, and release of Pol II into productive elongation, allowing rhythmicity both in recruitment and release rates (Fig 4C). A rigorous analytical and parameter-independent reanalysis of the model (S1 and S2 Texts, S1 Interactive Text) showed that two scenarios–a higher average Pol II recruitment rate, or a lowered release rate–could both explain the increased Pol II promoter-proximal pausing at SCPs compared to e.g. circadian type II promoters. The lower PI could in both these cases be explained by additionally assuming decreased transcriptional elongation speeds causing higher Pol II density (note that not transcriptional speeds, but rather transcriptional initiation rates determine how many transcripts are produced per time unit [66]). The first scenario, higher average Pol II recruitment rates, in theory generally comes with weaker combined net propagation of rhythms in Pol II recruitment and Pol II release rates to transcriptional activities (Fig 4C, S2 Text, S1 Interactive Text). Checking this prediction against data indeed revealed a negative correlation between PI and transcriptional amplitude (Spearman's rho = −0.30, p < 10^−15^). However, the assumption of only higher average Pol II recruitment rates for SCPs is not an attractive explanation for their high Pol II signals in the promoter-proximal regions, since SCPs drive strong rhythms. Instead, the situation for SCPs is better explained by invoking the alternative scenario (Fig 4C): Additionally assuming lowered average Pol II release rates also help explain the higher promoter-proximal Pol II signals of SCPs. This would be consistent with a tendency at SCPs for shifting of rhythmic control from Pol II recruitment to Pol II release, with retained overall rhythm propagation, that could be inferred from the data at hand (S1 and S2 Texts, S1 Interactive Text, S3E and S3F Fig).

If this is true, additionally a higher Pol II initiation rate (from recruited to paused Pol II) must be assumed to explain the high transcriptional activities of SCPs (S1 and S2 Texts, S1 Interactive Text). Such high initiation rates have indeed been found to be prevalent for TATA box promoters, partly due to a transcription initiation scaffold being constitutively bound to TATA box promoters of active genes [67]. A combination of much higher initiation rates and lower release rates would be consistent with all data: SCPs having higher promoter-proximal Pol II signal, lower PI, higher transcriptional rates, and stronger rhythms.

### Histone variant H2A.Z disfavors circadian rhythms

How might the predicted lower Pol II release rate be achieved? A possible mechanism could be depletion of the histone variant H2A.Z from the first (+1) nucleosome downstream of the TSS. Incorporation of the histone variant H2A.Z is recognized as a general route to transcriptional activation via various mechanisms [48]. In general, nucleosomes with the H2A.Z variant are more unstable, and the variant is associated with nucleosome-depleted regions [68,69], although the causal relationships behind this association are unclear [48]. Certain studies have also established a negative correlation between paused Pol II occupancy and H2A.Z. Possibly, H2A.Z at the first (+1) nucleosome downstream of the TSS facilitates the elongation of Pol II through the +1 nucleosome position [70]. If this is true, and if the average Pol II release rates of SCPs are indeed lower than for circadian type II promoters, then SCPs would have lower H2A.Z levels. If H2A.Z is associated with transcriptional activation, periodic up- and down-regulation might be incompatible with high H2A.Z levels. Hence, another question of more general nature is whether circadian transcriptional regulation is associated with H2A.Z depletion.

To address this, mouse liver H2A.Z ChIP-Seq data were reanalyzed. For all mouse promoters, the H2A.Z signal around the +1 nucleosome (determined from the MNase-Seq data with a peak detection algorithm, Methods) was computed, and normalized to the MNase-Seq based nucleosome signals. Indeed, there was a negative correlation between H2A.Z signal and levels of promoter-proximal paused Pol II for promoters with marked levels of paused Pol II (S3G Fig). In particular, SCPs were characterized by low H2A.Z levels and correspondingly high promoter-proximal paused Pol II levels. As was the case for Pol II coverage, the bootstrapping test procedure (Methods) showed that SCPs also have lower H2A.Z levels than highly expressed type I promoters (p < 10^−6^).

This is consistent with the model prediction that SCPs have lower Pol II release rates, given the notion that H2A.Z facilitates transcription through the +1 nucleosome. Depletion of H2A.Z might thus enable tighter regulation of Pol II release, exploited at promoters of genes transcribed in a strongly circadian manner. In line with the idea that H2A.Z might be more associated with constitutive transcription, there was a negative correlation in general between circadian transcriptional amplitudes and H2A.Z levels (Fig 4D, Spearman's rho = −0.28, p < 10^−15^).

### An H3K4me3 pattern extending into the gene bodies characterizes SCPs

The H3K4me3 modification is strongly associated with active transcription and has been implicated in generation of circadian rhythms in certain transcripts [26,71]. H3K4me3 is furthermore associated with high levels of Pol II in the promoter-proximal region [28], and might thus be a hallmark of SCPs. Inconsistent with this, however, is an observed positive correlation between H3K4me3 and H2A.Z levels [68]. Thus, there are reasons both to expect high as well as moderate H3K4me3 levels at SCPs. H3K4me3 levels of mouse liver promoters at different ZT have been analyzed by ChIP-Seq [10]. Although H3K4me3 levels oscillate at the promoters of many genes transcribed in a circadian fashion, the oscillations generally lag the transcriptional rhythms by several hours. Thus, rhythms in H3K4me3 levels near promoters might be consequences rather than causes of rhythmic transcription. This situation could, however, vary between promoter classes, although this possibility has yet not been investigated.

In order to clarify the role of H3K4me3 at SCPs and other promoter classes, the H3K4me3 ChIP-Seq data [10] were reanalyzed and quantified at different locations of the mouse promoters. This analysis showed that SCPs had significantly lower levels of H3K4me3 just downstream of the TSSs than circadian type II CTF binding promoters (Fig 4E, rank sum test, p = 3.8×10^−7^, median ratio 0.67). The lower H3K4me3 levels of SCPs apparently occur despite their higher promoter-proximal Pol II coverages: Normalizing each promoter-proximal Pol II level to its TSS H3K4me3 level (hereafter: P/M ratio) likewise resulted in significantly higher P/M ratios for SCPs compared to circadian type II CTF binding promoters (rank sum test, p < 10^−15^, median ratio 2.9). Again, the bootstrapping test procedure (Methods) verified that the TSS H3K4me3 levels and P/M ratios of SCPs indeed are significantly lower and higher, respectively, than for highly expressed type I promoters (p < 10^−6^ in both cases). This relationship apparently runs counter to the previously observed positive association between the H3K4me3 mark and promoter-proximal Pol II levels [28], which was reproduced with the present data set (S3H Fig). For a given promoter-proximal Pol II level, SCPs had atypically low H3K4me3 levels at the TSS. This does not mean that SCPs had very low H3K4me3 levels; in fact they were intermediate to high (Figs 4E and S3H), but significantly lower than expected given their extraordinarily strong promoter-proximal Pol II signals. Particularly notable, however, was the marked extension of the H3K4me3 mark far into the gene bodies of SCPs (Fig 4E), so that from a position of +500 bp of the TSS and onwards, genes with SCPs had the highest gene body H3K4me3 level of any group of genes, which was also verified by the bootstrapping test against expression-matched constitutive type I promoters (p < 10^−6^). There was also a general negative correlation between average TSS H3K4me3 levels and circadian transcriptional amplitudes (Spearman's rho = −0.37, p < 10^−15^), and consistent with this and the results regarding H2A.Z and amplitudes outlined above, the previously observed correlation between H2A.Z and H3K4me3 levels [68] was notable also in the present data set (S3I Fig). This suggests that although SCPs had intermediate to relatively high H3K4me3 levels, extremely high levels of the H3K4me3 mark do not go hand in hand with strong circadian rhythms. Previously, high H3K4me3 levels have been suggested to co-occur almost exclusively with high CpG ratios [45,54]. Although this correlation was evident also in the present data, SCPs constituted a notable exception, combining intermediate to relatively high H3K4me3 levels with low CpG ratios (S3J Fig).

A possible interpretation of these observations is that the observed intermediate H3K4me3 levels of SCPs reflect Pol II-mediated H3K4 trimethylation, pushing H3K4me3 levels up to intermediate rather than low levels. This intermediate level might be low enough to avoid a constitutively permissive chromatin state that would weaken rhythmic regulation. A non-causal role of H3K4me3 in circadian transcriptional activities has also been proposed to account for the later phases of rhythmic H3K4me3 levels with respect to transcriptional activities [10]. This would be consistent too with the high H3K4me3 levels in the gene body, since transcriptional elongation speed in the present analysis was predicted to be slower for genes with SCP promoters (see above), which could lead to the observed higher gene body Pol II signals. Indeed, the later phases of H3K4me3 signal compared to transcriptional activities were readily reproducible (S3K Fig) for CTF-binding promoters (including SCPs). However, this analysis revealed that rhythmic H3K4me3 levels of non-CTF binding circadian promoters tended to have earlier phases than those of transcriptional activities (S3K Fig). This opens up the possibility that H3K4 trimethylation plays a causal role in rhythmic transcriptional activation mediated by other TFs than CTFs.

### Shared characteristics of *Drosophila* and mouse circadian core promoters, but no *Drosophila* SCP class

The results reported so far established that mouse liver circadian promoters are strongly enriched for TATA box promoters, and further uncovered a special class of promoters: SCPs. These circadian promoter traits could be specific for mammals, or they could represent more universal constraints on core promoters for circadian regulation of transcription. A comparison with an evolutionary distant animal may throw light on the universality of circadian core promoters.

**Fig 5:**
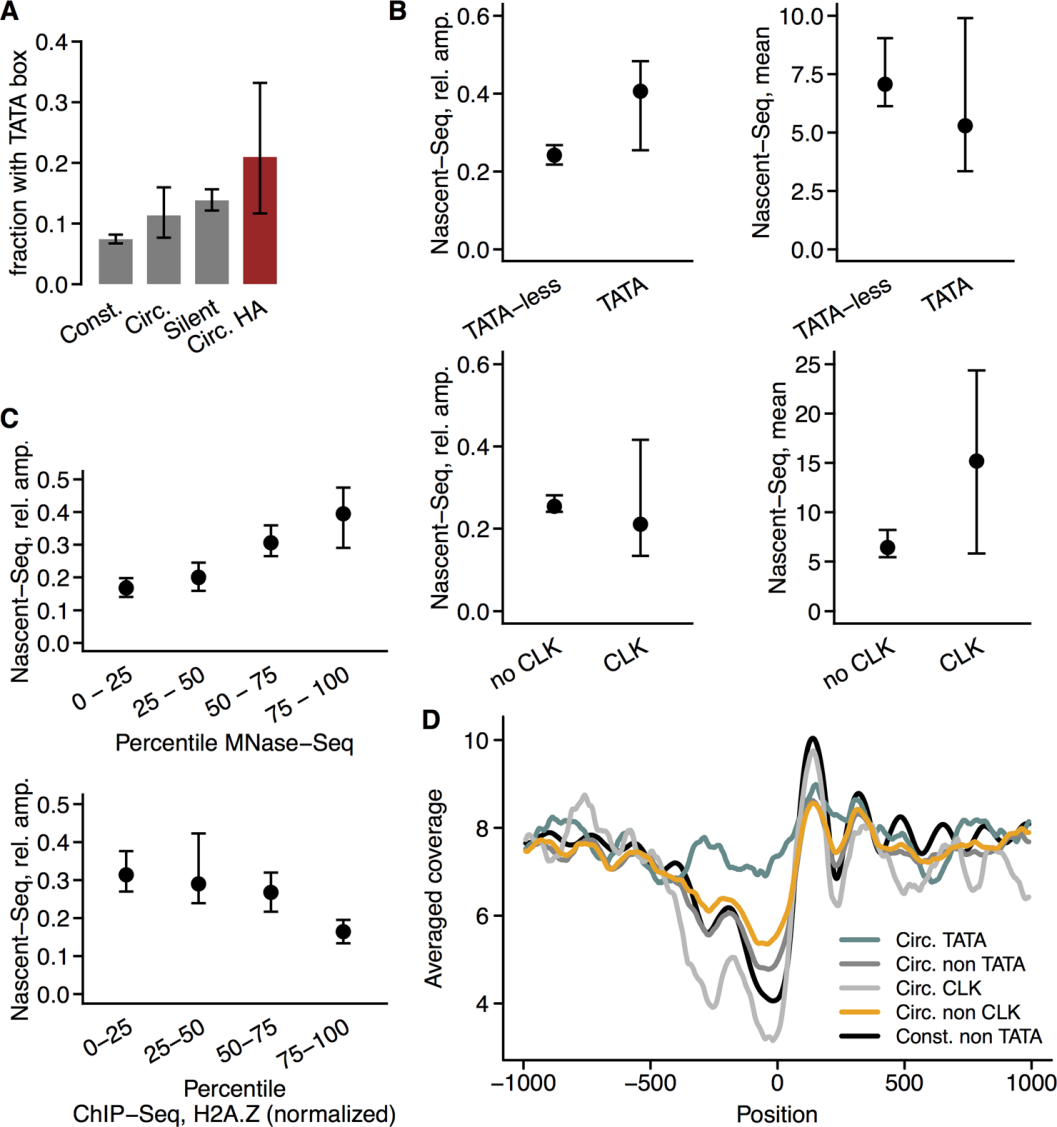
Properties of *Drosophila* circadian promoters. A. Fractions promoters with TATA box for different promoter classes. Legend: Const. = constitutive; Circ. = circadian; Circ. HA = circadian with high amplitude (upper 25% quantile). From left to right: n = 5134, 247, 1541, 62. B. Amplitudes and averages of transcriptional activities as measured by Nascent-Seq were quantified for the transcript corresponding to each promoter. Promoters were stratified as TATA box-containing and TATA-less (top), or as exhibiting either no, or at least one CLK ChIP-Seq peak (bottom). Medians and 95% confidence intervals are shown. From top to bottom, left to right: p = 0.0023, p = 0.16, p = 0.29, p = 0.066, rank sum tests. The last p value (mean for CLK vs. no CLK) became much smaller when considering all expressed transcripts: p = 2.5×10^−17^, median ratio 1.4 CLK vs. no CLK (S3 Table). C. Amplitude increases with nucleosome occupancy, and decreases with levels of nucleosome variant H2A.Z. Mean nucleosome pileups at positions 101 to 1 bp upstream of the TSS, and normalized H2A.Z levels at the +1 nucleosome peak, respectively, for all circadian promoters were ranked and divided into 4 quantiles. Amplitudes in each such quantile are visualized. D. Nucleosome occupancies around TSSs for different *Drosophila* promoter classes. Pileups were computed from MNase-Seq data (Methods) and averaged over the promoter classes for each position relative to the TSS (excluding top and bottom 1% values, respectively, due to a few outlier promoters). Circ. = circadian; Const. = constitutive.

*Drosophila* is besides mouse the model organism where the mRNA expression output pathways of the circadian clock have been best studied. Data from various studies of the *Drosophila* clock were compiled, resulting in a data set that, bar tissue-specificity, almost rivaled the data compiled for mouse liver. Time-resolved nascent and mature RNA-sequencing [12], and ChIP-Seq measurements for CLK binding sites [8] of fly head mRNA samples, nucleosome occupancies (MNase-Seq as well as H2A.Z ChIP-Seq), and transcribing Pol II positions in S2 cells (3' end nascent transcripts-sequencing, 3'NT-Seq) [70] as well as CAGE data on TSS usage in fly embryos [72] were compiled, using the RefSeq *Drosophila* transcript annotation (Methods). As was the case for mouse liver, *Drosophila* circadian transcripts turned out to be enriched for TATA boxes, compared to constitutive transcripts, especially transcripts with high amplitudes (Fig 5A, p = 0.035 and 0.00061, respectively, Fisher's exact test). Moreover, the presence of a TATA box positively correlated with amplitude but not mean mRNA expression levels, whereas CLK binding to the promoter region correlated with mean mRNA expression level but not amplitude (Fig 5B). These relationships are analogous to what was found for TATA box and CTF binding, respectively, in mouse liver. Reinforcing the analogy to mouse TATA box SCPs, circadian promoters were overall depleted of broad TSSs (Fisher's exact test, p = 0.0011, odds ratio 0.56). As for mouse liver, there was also a positive correlation between circadian amplitude and nucleosome occupancy immediately upstream of the TSS, as well as a negative correlation between transcriptional amplitude and H2A.Z content at the first nucleosome downstream of the TSS (Fig 5C, Spearman's rho = 0.44, p < 10^−12^ for nucleosome occupancy, Spearman's rho = −0.33, p = 1.2×10^−7^ for H2A.Z).

However, there were only 2 promoters both containing a TATA box as well as binding CLK. In fact, there was no evidence of a promoter class corresponding to mouse SCPs. Circadian promoters in *Drosophila* generally had high nucleosome occupancies (except CLK-binding promoters, Fig 5D), even circadian TATA box promoters driving strong average transcriptional rates (S4A Fig). Further, *Drosophila* circadian promoters driving high transcriptional amplitudes were depleted of paused Pol II compared to circadian promoters driving low transcriptional amplitudes (rank sum test, p = 8.8×10^−9^, median ratio 0.25). There was a negative correlation between paused Pol II and nucleosome occupancy immediately upstream of the TSSs of expressed transcripts (Spearman's rho = −0.20, p < 10^−15^), as previously described [30], which implicates competition between Pol II and nucleosome occupancy. However, there was no apparent SCP-like subpopulation of promoters with high amounts of paused Pol II and low nucleosome occupancy, at the same time also driving transcriptional rhythms with both high amplitudes and mean levels (S4A–S4D Fig).

These results suggest that the following traits: 1. high nucleosome occupancies, 2. low Pol II pausing levels, 3. depletion of the H2A.Z variant downstream of the TSS, and 4. a TATA box-based promoter architecture, are universally correlated to circadian regulation of transcription, albeit with moderate average mRNA expression levels. The evolution of SCPs–TATA box promoters with high Pol II pausing levels and low nucleosome occupancies that drive rhythms with both high averages and amplitudes–seems to not have occurred in *Drosophila*.

## Discussion

The results presented here show that the core promoter and its chromatin state are fundamental determinants of circadian transcriptional rhythms. This should motivate a broadening of focus for circadian transcriptional regulation studies to routinely cover aspects of the core promoter. Such efforts could also initially provide validation of the results of the present study. Further, when studying clock output mechanisms in cells or tissues, deletions of CTF binding sites, or over-expression of core clock genes, may abolish rhythmicity in expression of clock controlled genes, but also strongly influence average levels of gene product [26]. This makes it is difficult to discern whether observed phenotypes are due to loss of rhythmicity, or due altered average levels. By targeting the core promoter, e.g. the TATA box, by gene editing techniques [73,74], it might be possible to more precisely attenuate rhythmicity while keeping average levels constant.

Two major classes of circadian promoters emerged in the analysis. The most peculiar of these represented strong circadian promoters (SCPs), driving circadian transcription with both high amplitudes and high average rates. SCPs appear to represent a class of promoters that has not previously been characterized, combining traits of type I and type II core promoter classes as commonly defined [54], and combining circadian regulation of Pol II recruitment and pause release. With regard to chromatin state, a defining signature of SCPs was a high paused Pol II level combined with a low H3K4me3 level relative to the high Pol II level. On the other hand, SCPs had H3K4me3 levels extending further into the gene bodies than other promoters. Finally, although SCPs had low CpG ratios, they had marked nucleosome-depleted regions upstream of the TSSs. The second major circadian promoter class consists of type I promoters, which exhibited moderate average transcriptional activities and high nucleosome occupancies upstream of the TSSs.

The important role played by the core promoter may help explain why only some CTF binding promoters drive circadian transcription. The E-box is strongly associated with the generation of circadian rhythms in mammals and *Drosophila* since it provides a binding platform for circadian TFs CLOCK/BMAL1 and CLK/CYC, respectively. However, the E-box was originally discovered in the context of constitutive or transient activation of transcription, affecting processes involved in differentiation and development [75]. Thus, the E-box is presumably also implicated in non-rhythmic activation of target gene transcription. In the same way, ROR elements, which are the DNA recognition sites for nuclear receptors REV-ERB α and β, probably are able to bind other, non-rhythmic TFs of the nuclear receptor family, directing transient or constitutive rather than rhythmic transcription [76,77]. What decides whether a given CTF recognition site and promoter/transcript are associated with actual CTF binding and rhythmic transcription? The present results suggest that sequence features of the core promoter, such as TATA boxes, provide one piece of this puzzle. In particular, among circadian promoters, TATA boxes and low CpG content were more predictive of strong rhythms than CTF binding per se. Interestingly, a recent study found rather limited effects of BMAL1 deletion on CCG transcriptional rhythmicity in mouse liver [78].

What properties of the TATA box make it suitable for circadian regulation of transcription? TATA boxes can facilitate the assembly of a transcription reinitiation scaffold, consisting of TFIIA, TFIID, TFIIE, TFIIH, and Mediator, which remains on the promoter after Pol II promoter escape and makes rapid reassembly of the PIC possible [67,79]. Such reinitiation may not be unconditional, since the scaffold does not contain TFIIB and TFIIF, and since it is stabilized by TFs [79,80]. This scaffold mechanism involves TFIID, and although differentiated tissues in mammals do not always rely on TFIID for PIC formation [20,81], there is a subgroup of promoters in mouse liver that does show a strong dependence on TFIID for transcription [62]. Remarkably, CTF binding promoters and SCPs in particular accounted for around 89% of these TFIID-dependent promoters (S2E Fig). Thus, TATA box promoters and PIC scaffolds may constitute a suitable platform for strong but rhythmically gated transcription: in this scenario, circadian activators or repressors recruit TFIIB and Pol II in a rhythmic fashion to permanently assembled scaffolds.

The TFIIB-binding element BREd was underrepresented among circadian promoters. This element might thus rather be conducive to constitutive basal levels of TFIIB binding to promoters, especially in the scaffold scenario. TFIIB has been implicated in stimulating release of paused Pol II in via interactions with TFIIF [82–84], and constitutive TFIIB presence at the promoter may thus also dampen rhythms in release of paused Pol II. TFIIB binding might, in fact, be linked to high H3K4me3 levels [85]. Moderate H3K4me3 levels in the vicinity of the TSSs were indeed another hallmark of circadian promoters with high amplitudes (SCPs and circadian type I promoters). For SCPs, the levels were intermediate on an absolute scale but low given the high Pol II levels: high promoter-proximal Pol II levels and the H3K4me3 are generally associated [28,86]. There is considerable uncertainty as to the mechanistic causality relationships between the H3K4me3 mark and transcriptional activities [87]. Pol II can attract histone methylases leading to H3K4 trimethylation [42], but clearly, since SCPs had higher promoter-proximal Pol II than any other promoter class but lower H3K4me3 levels than circadian or constitutive type II promoters, other mechanisms must be at work. SCPs are CpG-poor, which may be a reason for their relative H3K4me3 depletion, since CpG dinucleotides may induce H3K4 trimethylation via CFP1 [45].

Why are promoters driving high amplitude circadian transcription (SCPs and circadian type I promoters) depleted of H3K4me3, relatively seen? Perhaps high levels of these nucleosome features should be viewed as hallmarks of high constitutive (or perhaps poised) transcription [32]. Circadian promoters need to not only rhythmically induce transcription, but also repress it, and possibly, slow kinetics of H3K4me3 demethylation precludes timely clearance of this histone mark at some promoters, so that they cannot drive circadian rhythms [10,58]. On the other hand, this might not be the case universally, since circadian type I promoters exhibited rhythmic H3K4me3 levels with earlier phases than transcription (S3K Fig), possibly indicating a causal role for this histone mark, as has also been demonstrated for certain genes [71]. Such a causal role may involve H3K4me3-mediated TFIID recruitment [43]. Assuming this scenario, a prediction is that H3K4me3 demethylation is only slow or inefficient enough to preclude circadian regulation when Pol II levels are constitutively high, perhaps persistently inducing H3K4 (re-)methylation. Finally, the extension of the H3K4me3 mark into the gene bodies of SCPs could be a by-product of high Pol II densities due to high rates of transcription initiation. This would also be in line with recent observations made by other investigators [88].

As was the case for the H3K4me3 mark, high levels of the H2A.Z nucleosome variant were also associated with lower amplitudes. These two observations are probably related, since the degree of correlation between the H3K4me3 mark and H2A.Z levels was notable, which corroborates earlier findings [38,68]. The association of H2A.Z with low amplitudes entails the prediction that regulation of H2A.Z levels at the time scale of circadian rhythms in general is not fast or reliable enough to be exploited for rhythm generation.

This may be the case for circadian nucleosomal occupancy dynamics in general. There are certainly cases where induced transcription involves nucleosome rearrangement [26,59,89]. However, the MNase-Seq data analyzed here provided no evidence at the population level of widespread rhythmic nucleosome occupancies at the promoters of circadian transcripts (sequencing depth was not high enough to reliably assess rhythms in nucleosome occupancy at single promoters). Absence of evidence is not evidence of absence, but notable is that rhythmic nucleosome occupancies at the population level was readily detectable at BMAL1 binding sites [60]. Thus, the data suggest that circadian core promoters have a relatively fixed nucleosome occupancy determined by factors not varying at the circadian time scale. This does not mean that nucleosome occupancy has nothing to do with transcriptional activities: nucleosome occupancies were negatively correlated to average transcriptional activities in a continuous fashion (S1F Fig). Such correlations are also present in yeast [90] and mouse and human embryonic stem cells [91].

Importantly, observed averages in tissue samples translate to probabilities of nucleosome occupancy in the single cell. There, nucleosomal occupancy is a dynamic phenomenon, where nucleosome sliding, binding and unbinding continuously takes place [92–94]. It thus makes sense that transcriptional activity varies continuously with nucleosome occupancy as observed at the tissue level, rather than being an on/off phenomenon. The average nucleosome occupancies may be set by a combination of sequence-determined nucleosome forming potential and auxiliary proteins [46,52]. However, these features do apparently not represent activities that vary in a circadian manner.

SCPs and circadian type I promoters have, by definition, low CpG ratios on average. This sequence feature is often considered to increase the nucleosome forming potential [50]. However, only circadian type I promoters had high nucleosome occupancies, those of SCPs were considerably lower (Fig 3C). The high average Pol II levels in the promoter-proximal regions may instead explain the low nucleosome occupancies, assuming competition between Pol II and nucleosome occupancy [46,53]. It is also possible that stable PIC scaffolds at the TATA boxes may compete with nucleosome formation. Thus, even though the low nucleosome occupancies of SCPs may appear to run contrary to the general association between high amplitudes and nucleosome occupancy (Fig 2C), their nucleosome forming potential is probably still high. It is notable that a high nucleosome forming potential and absence of permissive nucleosome features (H3K4me3, H2A.Z) were all associated with high circadian amplitudes. It appears that promoters with an active ground state are not generally employed to drive circadian transcription.

The establishment of a nucleosome-depleted chromatin state is apparently not widespread for *Drosophila* circadian promoters, where no counterpart of mouse SCPs was found. This could mean that SCPs are a relatively late evolutionary invention, and that CCGs originally employed the type I promoter architecture thought to be common for heavily regulated genes.

The short nascent mRNA produced by Pol II prior to entering the paused state might act as a recruitment platform for regulators of transcription [95]. Thus, future investigations of circadian recruitment regulators of pause release at SCPs may also be directed to nascent mRNA interacting proteins. More broadly, investigators of clock-controlled genes may find the specific signatures of SCPs or type I circadian promoters useful to direct experimental designs when studying mechanisms of transcriptional regulation.

## Methods

### Promoters and CAGE data

Mouse promoters were compiled from the UCSC mm9 RefSeq annotation [96,97]. Then, CAGE clusters were computed based on the "FANTOM3and4"/"FANTOMtimecourseCAGEmouse"/"liver_under_constant_darkness" data set as provided by the CAGEr/FANTOM3and4CAGE R packages [57], using the recommended procedures in the package. CAGE peak widths were computed for locations ±300 bp of the TSSs provided by the UCSC RefSeq annotation, using the tagClusters function; the widths were defined as the distance between the 10% and 90% quantiles. The CAGE clusters represent mouse liver TSSs; for the cases that a CAGE cluster was measured within 300 bp of a RefSeq-annotated TSS, the CAGE location was used as a TSS instead, resulting in slight adjustments of 14585 out of 33333 RefSeq transcript TSSs. The distribution of CAGE peak widths was bimodal (S2D Fig), prompting a classification of promoters with a CAGE peak width smaller than 10 as focused, otherwise as dispersed.

Then, transcript sets with identical start and end coordinates were reduced to all but one transcript, leaving 27274 transcripts. Further, for TSSs corresponding to more than 1 resulting transcripts, only the shortest transcript was kept, 26251 transcripts now remained. Finally, a set of transcripts unambiguously assignable to a TSS was needed to conduct the present study, since it combines core promoter and transcript properties. For this, any overlapping transcripts with different TSSs needed to be discarded. Finally, transcripts shorter than 500 bp and non-protein coding transcripts (RefSeq annotation starting with "NR") were excluded, leaving a set of 17686 promoters and transcripts used for the following analysis.

*Drosophila* promoters were compiled from the UCSC dm3 refGene table, which corresponds to the FlyBase transcript annotation [98]. The same procedure as for the mouse genome (above) resulted in 11096 *Drosophila* promoters.

*Drosophila* CAGE peaks classified as "peaked" (= focused) or "broad" (= dispersed) were obtained from a study of fly embryos [72]. The nearest CAGE peak within 300 bp of the UCSC refGene TSS annotation (if there was such a peak) was used to classify TSSs.

### Nascent-Seq, RNA-Seq, and microarray data

Mouse liver Nascent-Seq and RNA-Seq data from a study of mice kept under 12 hr/12 hr light-dark cycles [11] were obtained from the NCBI sequence read archive, accession numbers SRP011984 and SRP011981, respectively. Reads were aligned to the UCSC mm9 assembly using the Bowtie2/TopHat2 pipeline [99,100], allowing only uniquely mapped reads. Duplicate reads were removed. Transcripts were quantified against the USCS mm9 RefSeq annotation by counting reads mapping anywhere within the exons for Nascent-Seq, or reads compatible to splicing annotation for RNA-Seq; the R package GenomicAlignments [101] was used to design this workflow. Transcript abundances were reported as standard normalized RPKM values, with a further correction step using the calcNormFactors function of the R package edgeR [102].

Microarray data from samples of livers of mice kept under similar light-dark conditions [63] were obtained from NCBI GEO, accession number GSE33726, RMA normalized and summarized according to the RefSeq annotation using Brainarray v. 18 CDF files [103].

*Drosophila* head Nascent-Seq and RNA-Seq data [12] were obtained from the NCBI sequence read archive, accession number SRP012175. Transcript abundances were quantified using the same workflow outlined above.

### Circadian rhythms detection

Circadian rhythms in nascent or mature transcript abundances were detected using the RAIN algorithm and R package [104]. For both mouse liver and *Drosophila* heads, data spanned two days, with samples taken every 4 hrs, yielding 12 samples for each Nascent-Seq or poly(A)^+^ RNA-Seq data set. Averages and relative amplitudes were quantified by harmonic regression using the HarmonicRegression R package [13]. Expressed transcripts were defined as transcripts having mean nascent RPKM values > 0.1 (mouse) or > 1 (*Drosophila*). Silent transcripts were defined as having mean nascent RPKM values of < 0.01 in both organisms. Mouse circadian transcripts were defined as expressed transcripts having Benjamini-Hochberg corrected RAIN p values < 0.2 and circadian amplitudes > 0.1 as estimated by harmonic regression. *Drosophila* circadian transcripts were defined as expressed transcripts having Benjamini-Hochberg corrected RAIN p values < 0.25 (the laxer constraints on *Drosophila* transcripts were necessary in order to obtain a number of circadian transcripts large enough for reliable statistics). Highly expressed transcripts were defined as the upper 25% quantile of mean transcriptional activities of expressed transcripts. High amplitude transcripts were defined as the upper 25% quantile of transcriptional amplitudes of circadian transcripts.

### Detecting sequence features

Position count matrices (PCM) for the TATA box, BREu, BREd DNA sequences were obtained from JASPAR [105]. To discover matches for these matrices, standard logarithmic odds scores for each matrix were computed [106], corresponding to the GC content of each promoter. A rigorous method [107] was used to set score thresholds for each matrix and promoter search region. This method computes the false discovery (FDR) and false negative rates (FNR) of discovery for a PCM and a DNA sequence, given the sequence's GC content; here, the threshold that brings the FDR and FNR as close as possible was used to determine hits. Further, a rigorous method for PCM regularization [107] was used on the JASPAR matrices as a preprocessing step. These algorithms were implemented as the accompanying R/Bioconductor package "profileScoreDist" (https://bioconductor.org/packages/profileScoreDist). The package is generally applicable to any position count matrices.

Scans were made for TATA box PCM matches between −50 (start position) and −10 bp (end position) of mouse and *Drosophila* TSSs, and when plotting the positions of all mouse matches (not just the one closest to the *a priori* consensus position [108]), the familiar TATA box peak at around position −30 of the TSS [109] was recovered (S1A Fig), which validated the present approach. The BREu and BREd PCMs were scanned for between the −75/−26 and the −30/−1 positions, respectively. Additionally filtering for evolutionary conservation improves *Drosophila* DNA binding site predictions, but this effect is less clear for mouse [110]. Hence, only *Drosophila* TATA box hits with a median phastCons score > = 0.75 were retained. The PhastCons scores [111] were obtained from UCSC for the *Drosophila melanogaster* dm3 assembly, median phastCons scores were computed for each TATA box hit. For the promoters with more than one TATA box hit (these were often overlapping hits), the maximal median phastCons score was reported.

CpG ratios (observed/expected CG dinucleotides) were determined for mouse promoters using the standard formula CG×N/(G×C) [112], where N is the width of the DNA sequence considered. Here, an interval between −100 and +100 of the TSS was scanned for the CpG ratio determination, so that N = 200 (by convention, there is no "0" position).

### CTF ChIP-Seq data

Mouse liver ChIP-Seq peaks for BMAL1 (E-box binding) [4] were obtained from the Supplementary file doi:10.1371/journal.pbio.1000595.s019. Normalized tag counts for each time point as given in this file were used to estimate BMAL1 binding phases (ZT) using the HarmonicRegression package.

Mouse liver ChIP-Seq peaks for REV-ERB α and β (ROR element binding) [5] were obtained from GEO (accession numbers GSM840528 and GSM840529, respectively).

Mouse liver ChIP-Seq peaks for E4BP4 (D-box binding) [6] were obtained from GEO (accession number GSM1437733). All mouse liver ChIP-Seq peaks were from reads aligned to the mouse mm9 assembly.

*Drosophila* ChIP-chip calls for CLK (E-box binding) [8] were obtained from the Supplementary Table 1 of that article, cycling peaks were retained. The chip used was based on the *Drosophila* dm3 (BDGP5) assembly.

ChIP-Seq and ChIP-chip peaks were narrowed to their center coordinates, which were matched to regions ±3000 of the set of TSSs, except for *Drosophila* CLK peaks, which were matched to ±2000 bp regions around the TSSs, following the original study. Promoter were classified as CTF binding if they had one or more CTF peak center within these regions.

### Mouse liver Pol II and H3K4me3 ChIP-Seq data

ChIP-Seq reads were aligned to the mouse mm9 genome assembly using the BWA aligner [113]. Duplicate reads were removed, and aligned reads with a phred alignment quality of 30 or greater were retained.

Reads were mapped to the promoter regions using the GenomicAlignments package [101]; the "coverage" function was used to compute pileups for each promoter: reads per base per million reads (e.g. Fig 4A). Reads were the shifted equal amounts for top and bottom strands to maximize the correlation between coverages at both strands for regions spanning −500 to +500 of all TSSs, resulting in shifts of 35–40 bp for each strand, as in the previous study [10]. The bimodal peaks evident in the Pol II pileups were similar to those observed earlier [32]. Pileups were averaged over the 7 time points of the study. For the final pileup averaging across promoter groups (e.g. Fig 4A), top and bottom 1% quantiles were left out of the averaging, due to a few outliers.

For statistical tests and calculation of circadian phases (ZT), mouse liver promoter-proximal pausing region Pol II levels were quantified for each of the 7 time points as RPKM counts of ChIP-Seq reads overlapping the region +21 to +100 bp of the TSS. Gene body Pol II was quantified as RPKM counts of reads overlapping the positions +301 to +1300 bp downstream of the TSS for transcripts 1300 bp or longer, otherwise between positions +301 and +500 for the few short transcripts. PIs were computed as proximal/gene body Pol II signals.

Promoter-proximal (TSS) H3K4me3 (ChIP-Seq data spanning the same 7 time points as the Pol II data) was quantified in the same way using the +1 to +200 bp interval, downstream gene body H3K4me3 was quantified using the +801 to +1000 bp interval.

As in the original study [10], Pol II and H3K4me3 values were first quantile normalized over the set of promoters, then the R package HarmonicRegression [13] was used to compute phases (ZT) and means based on the 7 time points. This yielded ZT estimations with reasonable confidence intervals, and also p values against the null hypothesis of random signals without rhythms. For 7 time points, no rigorous false discovery rate cutoffs can be applied for thousands of promoters. Rather, to compute phase differences, promoters with HarmonicRegression rhythm p values < 0.1 for all thee of promoter-proximal Pol II, gene body Pol II, and PI, were compiled, and phase differences computed for S3E Fig.

### MNase-Seq, H2A.Z ChIP-Seq, and GRO-Seq data

Aligned and normalized mouse liver MNase-Seq and H2A.Z Chip-Seq reads for livers of WT and BMAL1^−/−^ knockout mice [60] were obtained as bigWig files from GEO, accession numbers GSE47142 and GSE47143, respectively. Mouse liver GRO-Seq data were obtained as bigWig files from GEO, accession number GSE59486 [6]. Pileups (reads per bp per ten million reads) were computed with the GenomicAlignments R package for each time point, and then averaged over time points (mean) for all analyses except the nucleosome rhythmicity analysis. For the final pileup averaging across promoter groups (e.g. Fig 4B), top and bottom 1% quantiles were left out of the averaging, due to a few outliers.

For statistical tests, mean nucleosome pileups for the regions between −101 and −1 bp of the TSS were averaged to represent nucleosomal coverages.

The locations of the first (+1) nucleosome peak downstream of the TSSs in the pileups were detected exactly as described in the original report [70]. The procedure was implemented in the accompanying R package peakPick (https://cran.r-project.org/web/packages/peakPick/index.html).

H2A.Z and nucleosomal pileups were averaged over intervals between −80 and +80 bp of the +1 peaks, then the H2A.Z signals were normalized to the nucleosomal signals. This value was used as the H2A.Z signal for downstream analysis.

### *Drosophila* MNase-Seq, Pol II and H2A.Z ChIP-Seq data, and 3'NT-Seq data

*Drosophila* S2 cell MNase-Seq, H2A.Z ChIP-Seq, and 3'NT-Seq data [70] were obtained in the wiggle format from GEO, accession number GSE49106. Pileups for each promoter were created exactly as described for the mouse liver MNase-Seq and ChIP-Seq data, except that the normalization as given in the wiggle files was retained.

For statistical tests, averaged nucleosome pileups for the regions between −100 and −1 bp of the *Drosophila* TSSs were averaged to represent nucleosomal coverages.

The locations of the first (+1) nucleosome peaks and the H2A.Z signals normalized to the MNase-Seq signal for these +1 peaks were computed exactly as for the mouse liver TSSs described above.

For statistical tests, *Drosophila* Pol II promoter-proximal pausing region signals were quantified as 3'NT-Seq normalized pileups averaged over the region +1 to +100 bp of the TSS. Gene body Pol II signals were quantified as normalized pileups averaged over the region +250 –+650 bp downstream of the +1 nucleosome. Due to a few outliers, top and bottom 1% quantiles were left out of the averaging. Finally, *Drosophila* PIs were computed as the ratios between these two signals. The original study [70] analyzed the specific phenomenon of Pol II stalling at the +1 nucleosome position, a phenomenon distinct from regulated pausing. These results were reproduced and related to the PI estimation used here (S5 Fig).

### SCP bootstrapping test procedure

Mean transcriptional activities were binned into 55 bins spanning the logarithmic scale. Non-CTF binding constitutive promoters with TATA box (constitutive type I) had probabilities assigned to each bin. These probabilities were weighted according to the counts of SCPs with transcriptional activities falling within the corresponding bin, then normalized. This enabled sampling of non-CTF binding constitutive promoters to obtain populations with the same mean transcriptional activity distributions as SCPs. Properties of SCPs compared to those of the sampled population of "non-SCPs with SCP-like transcriptional activities" could then be analyzed to distinguish SCP features that are not merely epiphenomena of high transcriptional activities.

For this, standard bootstrapping procedure was employed: The SCP properties and constitutive type I promoter properties to compare (such as CpG ratio) and their probability distributions (uniform for SCPs, weighted probabilities as outlined above for constitutive type I promoters) were pooled, samples of the same size as the SCP group and the constitutive type I promoters group, respectively, were drawn randomly with replacement from the pool, and a rank sum test statistic was computed each time. This was done 1,000,000 times. The resulting empirical test statistic probability distribution was then used when repeatedly drawing samples from each population separately, each time with sample sizes corresponding to the population sizes, rank sum test statistics and median location differences were computed. Then, two-sided p values could be estimated; 1000 such p values were computed for each property, median p value was reported (S2 Table).

### TFIID-dependent transcripts

Gene symbols for up- (at least 2-fold) and down-regulated (at least 0.5-fold) transcripts at Postnatal day 30 (P30) in livers of *Taf10^lox/lox^-AlbCre* mice were obtained from Supplementary Table 1 of the original article [62], then matched to the promoter collection used in the present work. These mice experience a liver-specific deletion of the *Taf10* gene postnatally between days P15 and P22.

## Supporting Information

S1 Fig A. Positions of the first "T" of all TATA box position count matrix hits in all mouse promoters scanned. B. Transcriptional amplitudes (Nascent-Seq data, Methods) plotted against CpG ratios for all circadian promoters. Spearman's rho = −0.24, p < 10^−15^. A least squares regression line is plotted to highlight the negative correlation. C. Low CpG ratios are associated with high amplitudes. The overall bimodal CpG ratio distribution evident in panel D below motivated the binary classification around CpG ratio = 0.5. Amplitudes were computed for all circadian promoters, and promoters with high CpG ratios had significantly lower amplitudes (rank sum test, p < 10^−15^, median ratio 1.34). Median and 95% confidence intervals are visualized. D. CpG ratio distributions. CpG ratios were stratified according to promoter class. Data are presented as kernel densities, which are smooth analogues to histograms computed with the standard R kernel density algorithm. E. Nucleosome occupancies (MNase-Seq data, Methods) immediately upstream of the TSSs plotted against CpG ratios for all promoters corresponding to expressed transcripts. Spearman's rho = −0.45, p < 10^−15^. F. Mean transcriptional activities (Nascent-Seq data, Methods) plotted against nucleosome occupancies (as in panel C) for promoters corresponding to all expressed genes.
(PDF)

S2 Fig A. Amplitudes and averages of transcriptional activities as measured by Nascent-Seq were quantified for the transcript corresponding to each promoter. Stratifications were made either according to TATA box presence in the promoter, or according to CpG ratio. B. Nucleosome occupancies immediately upstream of the TSSs plotted against CpG ratios for all circadian promoters (upper panel). SCPs (red dots) had on average lower nucleosome occupancies than other circadian promoters. This effect dominates for low CpG ratios, as evident in the lower panel, where medians and 95% confidence intervals are shown for given CpG ratio intervals. C. Nucleosome occupancies around TSSs for SCPs, as measured in liver samples from wild type and BMAL1^−/−^ mice, respectively. SCPs were here limited to those SCPs with BMAL1 ChIP-Seq peaks, in order to show that these promoters still have low nucleosome occupancy upstream of the TSSs. Pileups were computed from MNase-Seq data (Methods) and averaged over the promoters for each position relative to the TSS (excluding top and bottom 1% values, respectively, due to a few outlier promoters). BMAL1 SCPs = SCPs with at least one BMAL1 ChIP-Seq peak. D. CAGE peak width distributions (Methods) for different promoter classes. Const. = Constitutive. E. Fractions of TFIID-dependent promoters (Methods), and other promoters driving expressed transcripts, respectively, with TATA boxes or LCpG and CTF ChIP-Seq peaks, respectively (since not only circadian transcripts were considered, TATA box/LCpG promoters are not called SCPs here). Error bars represent 95% confidence intervals assuming binomial distributions. Bar groups: *n* = 10899 and 130, respectively. F. Reproduction of Fig 3E, but with transcription abundances obtained from mouse liver microarray data (Methods). Error bars represent 25% and 75% quantiles, respectively. Abbreviations as for panel B.
(PDF)

S3 Fig Nucleosome, Pol II, and H3K4me3 characteristics of circadian promoters. Note that the RPKM Pol II and H3K4me3 values given in the scatter plots here are not equivalent but proportional to the per-base pileup values in Fig 4. See Methods for details. "Other" refers to circadian promoters other than SCPs. A. Averaged pileups of GRO-Seq reads around the TSSs for different promoter classes. Note the "cliff" at around position +150; reads were extended from 50 to 150 bp [6], so that this cliff marks the characteristic pausing position ~50 bp downstream of the TSS. Circ. = Circadian; Const. = Constitutive; Type I = non-CTF binding circadian promoters with TATA box or LCpG, type II = CTF binding circadian promoters without TATA box and with HCpG. B. Levels of Pol II in the promoter-proximal pausing region plotted against averaged nucleosome pileups at between −101 and −1 bp of the TSSs. C. PI plotted against nucleosome pileups as in panel A. D. Oscillations in nucleosome and Pol II pileups as in Fig 4B, but for other promoter groups. E. Phase signatures of circadian Pol II recruitment and release. Left panel: Circles represent phases of paused Pol II, gene body Pol II, and pausing index, respectively. Only a combination of circadian regulation of recruitment and pause release results in paused and gene body Pol II with the same phase, at the same time with paused Pol II and PI with opposite phases (middle column). Not shown is the transition from closed to open DNA-Pol II complex that is also part of the model (S2 Text). Right panel: Estimated phase differences between paused Pol II and gene body Pol II (Δ ZT_gb_), and between paused Pol II and PI (Δ ZT_PI_), respectively; details of computation method are given in the Methods section. The observed combination of small Δ ZT_gb_ phase differences and large Δ ZT_PI_ phase differences is only compatible with combined circadian regulation of Pol II recruitment and pause release. F. Fractions of different circadian promoter classes with rhythmic PI. Harmonic regression p values for PI oscillation were computed for each promoter (Methods), and the fractions of rhythmic promoters in each promoter group for different p value cutoffs are plotted. SCPs exhibit larger fractions independently of cutoff. Abbreviations as for panel A. G. Levels of Pol II in the promoter-proximal pausing region plotted against normalized H2A.Z levels at the +1 nucleosome (Methods). A population of promoters with both low Pol II levels and H2A.Z levels may be contrasted against a population of promoters with higher Pol II and H2A.Z levels. SCPs are mainly found in the population with higher levels. For that population (here defined as having paused Pol II greater than 10), there was a negative correlation between paused Pol II and H2A.Z levels (Spearman's rho = −0.32, p < 10^−15^). H. Promoter-proximal H3K4me3 levels plotted against levels of Pol II in the pausing region. I. Promoter-proximal H3K4me3 levels plotted against H2A.Z levels at the +1 nucleosome. J. Promoter-proximal H3K4me3 levels plotted against CpG ratios. K. Phase differences between transcriptional rhythms (as estimated from Nascent-Seq data) and promoter-proximal H3K4me3 rhythms, as estimated from H3K4me3 ChIP-Seq data (Methods). Positive phase differences mean that the H3K4me3 level has an earlier phase than transcriptional activity. Circ. = Circadian promoters with, in addition, rhythmic H3K4me3 levels (harmonic regression p value < 0.1). Data are presented as kernel densities. Phase differences were different for CTF (circular mean: −0.9 hrs) and non CTF binding promoters (circular mean: +3.0 hrs, Watson's two-sample test for circular data, p < 0.01).
(PDF)

S4 Fig No apparent SCP counterpart in *Drosophila*. Distributions of nucleosome occupancies, promoter-proximal pol II levels, and transcriptional activities hint at the SCP population in mouse liver, but not in *Drosophila*. A. Nucleosome occupancies immediately upstream of the TSSs (Methods), stratified for circadian TATA box promoters associated with highly expressed (upper 25% quantile, Methods) transcripts and the other 75%, respectively. All *Drosophila* circadian promoters, including highly expressed had high nucleosome occupancies, compared to highly expressed constitutive promoters, which had low nucleosome occupancies. On the other hand, in a corresponding plot for mouse liver circadian promoters, the SCP TATA box population is visible with its low nucleosome occupancies. Circ. = Circadian, Const. = Constitutive. B. Comparing nucleosome occupancies immediately upstream of the TSSs for circadian promoters with TATA boxes reveals a subpopulation of mouse promoters with low nucleosome occupancies, but not for *Drosophila* promoters. Kernel densities of MNase-Seq data (Methods) are visualized. C. For mouse circadian promoters with TATA box, there is a visible subpopulation with high transcriptional activities. Such a subpopulation is not apparent for *Drosophila* promoters. Kernel densities of Nascent-Seq data (Methods) are visualized. D. For mouse circadian promoters with TATA box, there is a visible subpopulation with high Pol II occupancies in the pausing region immediately downstream of the TSSs (Pol II ChIP-Seq data, Methods). Such a subpopulation is not apparent for *Drosophila* promoters (3'NT-Seq data, Methods).
(PDF)

S5 Fig With the promoter selection made in the present work, the results from the earlier study [70] still hold. Stall fractions (SFs) measure the percentage of positions with stalled Pol II in the 100 bp upstream of the first nucleosome downstream of the TSS. SFs and nucleosome positions were computed exactly (as far as possible) according to the description in the original article: Stalled positions are defined as 3' end nascent seq peaks rising significantly above their immediate surroundings. The algorithms (SF computation and nucleosome peak detection) were incorporated into the R package "peakPick" (Methods). A. Promoters were classified as having high (SF > 0.6) or low (0.05 < SF ≤ 0.3) SFs. Low SF promoters exhibit slightly lower nucleosome occupancies, which can be interpreted that stalled Pol II is associated with nucleosome barriers. B. Low SF promoters exhibit higher H2A.Z levels (here not normalized to bulk nucleosome levels), which may be interpreted as H2A.Z being associated with less stalling. C and D. Expressed transcripts were binned along SF intervals 0.1–0.3, 0.3–0.5, 0.5–0.7, and 0.7–1. Averaged normalized H2A.Z levels (C) and nucleosome levels (D) at the +1 nucleosome peak are plotted for each bin. Due to outliers, 20% top and bottom values were left out of the averaging for the H2A.Z levels. Error bars refer to SEM. The increased +1 nucleosome levels associated with high SFs must not be confused with the negative correlation between PI and nucleosome occupancies immediately upstream of the TSSs, demonstrated with the same data set as outlined in the main text. E. PI is correlated to SF. For promoters with PI and SF greater than 0, these two measures of Pol II pausing and stalling, respectively, were correlated (Spearman's rho = 0.32, p < 10^−15^). A standard linear least squares regression line visualizes the correlation.
(PDF)

S1 Table Definitions and properties of SCPs and promoters of all expressed transcripts, respectively, for mouse liver (Methods). (XLSX)

S2 Table Summary of the results of the bootstrapping tests. (XLSX)

S3 Table Definitions and properties of promoters of all expressed transcripts for *Drosophila* (Methods). (XLSX)

S1 Text Overview of computational analysis of rhythm propagation from Pol II recruitment and release, respectively, to transcriptional activity. (PDF)

S2 Text Detailed mathematical analysis of rhythm propagation from Pol II recruitment and release, respectively, to transcriptional activity. (PDF)

S1 Interactive Text *Mathematica* notebook for analysis of rhythm propagation from Pol II recruitment and release, respectively, to transcriptional activity. (NB)
