# Supplementary figures and images for: Linking Core Promoter Classes to Circadian Transcription
Source: PLoS Genet. 2016 Aug 9;12(8):e1006231. doi: 10.1371/journal.pgen.1006231 (PMC4978467; doi:10.1371/journal.pgen.1006231)

**A**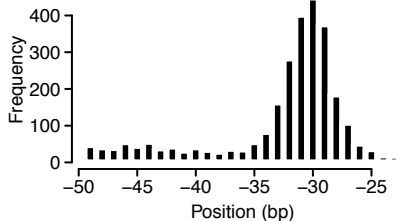**B**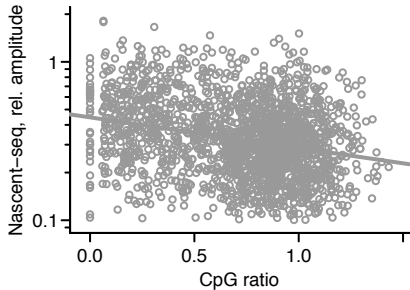**C**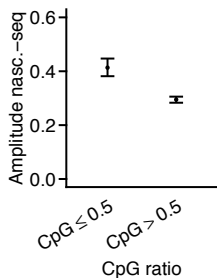**D**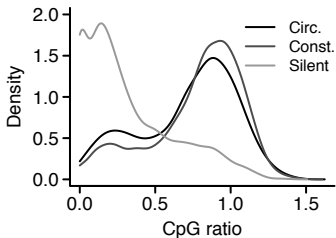**E**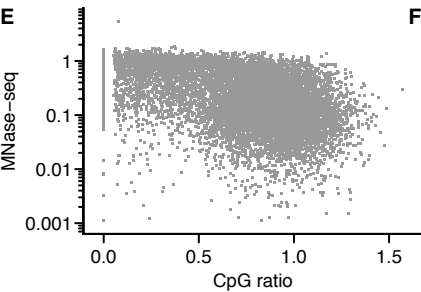**F**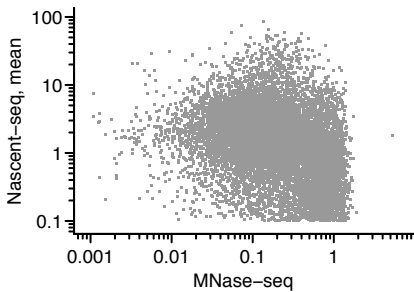

Supplement: S1 Fig — A. Positions of the first "T" of all TATA box position count matrix hits in all mouse promoters scanned. B. Transcriptional amplitudes (Nascent-Seq data, Methods) plotted against CpG ratios for all circadian promoters. Spearman's rho = −0.24, p < 10−15. A least squares regression line is plotted to highlight the negative correlation. C. Low CpG ratios are associated with high amplitudes. The overall bimodal CpG ratio distribution evident in panel D below motivated the binary classification around CpG ratio = 0.5. Amplitudes were computed for all circadian promoters, and promoters with high CpG ratios had significantly lower amplitudes (rank sum test, p < 10−15, median ratio 1.34). Median and 95% confidence intervals are visualized. D. CpG ratio distributions. CpG ratios were stratified according to promoter class. Data are presented as kernel densities, which are smooth analogues to histograms computed with the standard R kernel density algorithm. E. Nucleosome occupancies (MNase-Seq data, Methods) immediately upstream of the TSSs plotted against CpG ratios for all promoters corresponding to expressed transcripts. Spearman's rho = −0.45, p < 10−15. F. Mean transcriptional activities (Nascent-Seq data, Methods) plotted against nucleosome occupancies (as in panel C) for promoters corresponding to all expressed genes. (PDF) [file pgen.1006231.s001.pdf]

**A**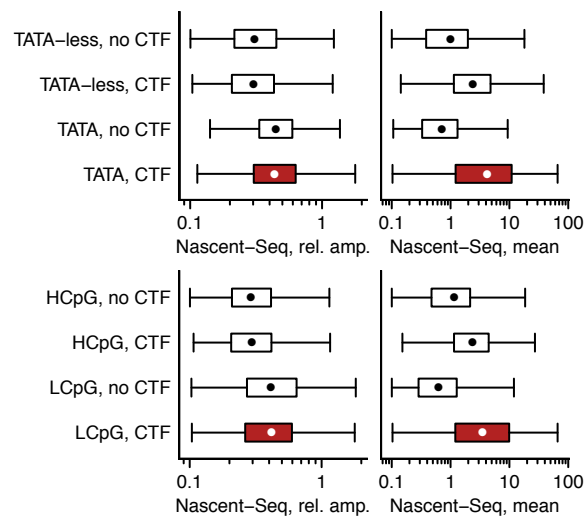**B**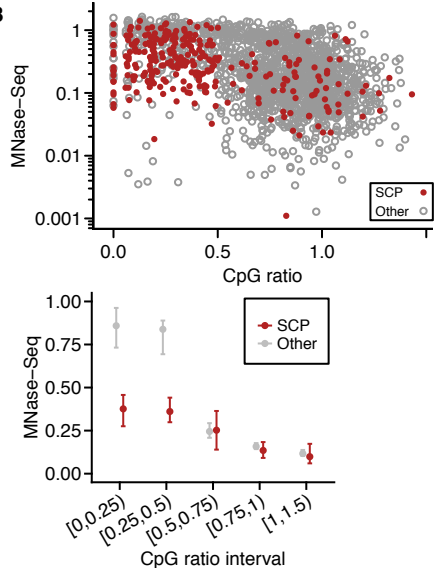**C**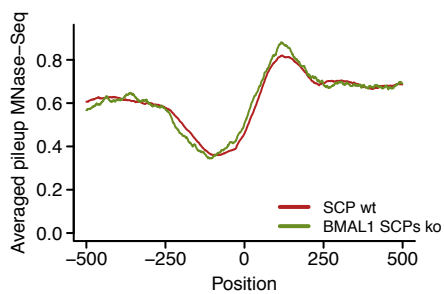**D**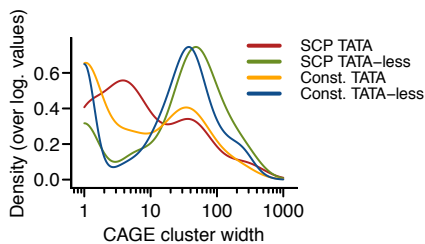**E**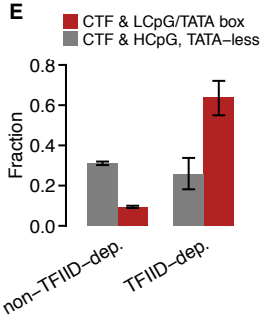**F**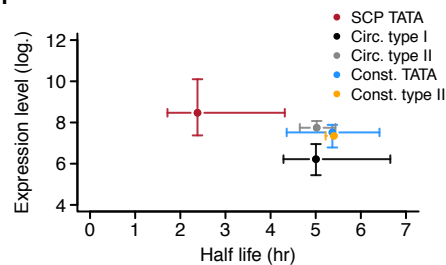

Supplement: S2 Fig — A. Amplitudes and averages of transcriptional activities as measured by Nascent-Seq were quantified for the transcript corresponding to each promoter. Stratifications were made either according to TATA box presence in the promoter, or according to CpG ratio. B. Nucleosome occupancies immediately upstream of the TSSs plotted against CpG ratios for all circadian promoters (upper panel). SCPs (red dots) had on average lower nucleosome occupancies than other circadian promoters. This effect dominates for low CpG ratios, as evident in the lower panel, where medians and 95% confidence intervals are shown for given CpG ratio intervals. C. Nucleosome occupancies around TSSs for SCPs, as measured in liver samples from wild type and BMAL1−/− mice, respectively. SCPs were here limited to those SCPs with BMAL1 ChIP-Seq peaks, in order to show that these promoters still have low nucleosome occupancy upstream of the TSSs. Pileups were computed from MNase-Seq data (Methods) and averaged over the promoters for each position relative to the TSS (excluding top and bottom 1% values, respectively, due to a few outlier promoters). BMAL1 SCPs = SCPs with at least one BMAL1 ChIP-Seq peak. D. CAGE peak width distributions (Methods) for different promoter classes. Const. = Constitutive. E. Fractions of TFIID-dependent promoters (Methods), and other promoters driving expressed transcripts, respectively, with TATA boxes or LCpG and CTF ChIP-Seq peaks, respectively (since not only circadian transcripts were considered, TATA box/LCpG promoters are not called SCPs here). Error bars represent 95% confidence intervals assuming binomial distributions. Bar groups: n = 10899 and 130, respectively. F. Reproduction of Fig 3E, but with transcription abundances obtained from mouse liver microarray data (Methods). Error bars represent 25% and 75% quantiles, respectively. Abbreviations as for panel B. (PDF) [file pgen.1006231.s002.pdf]

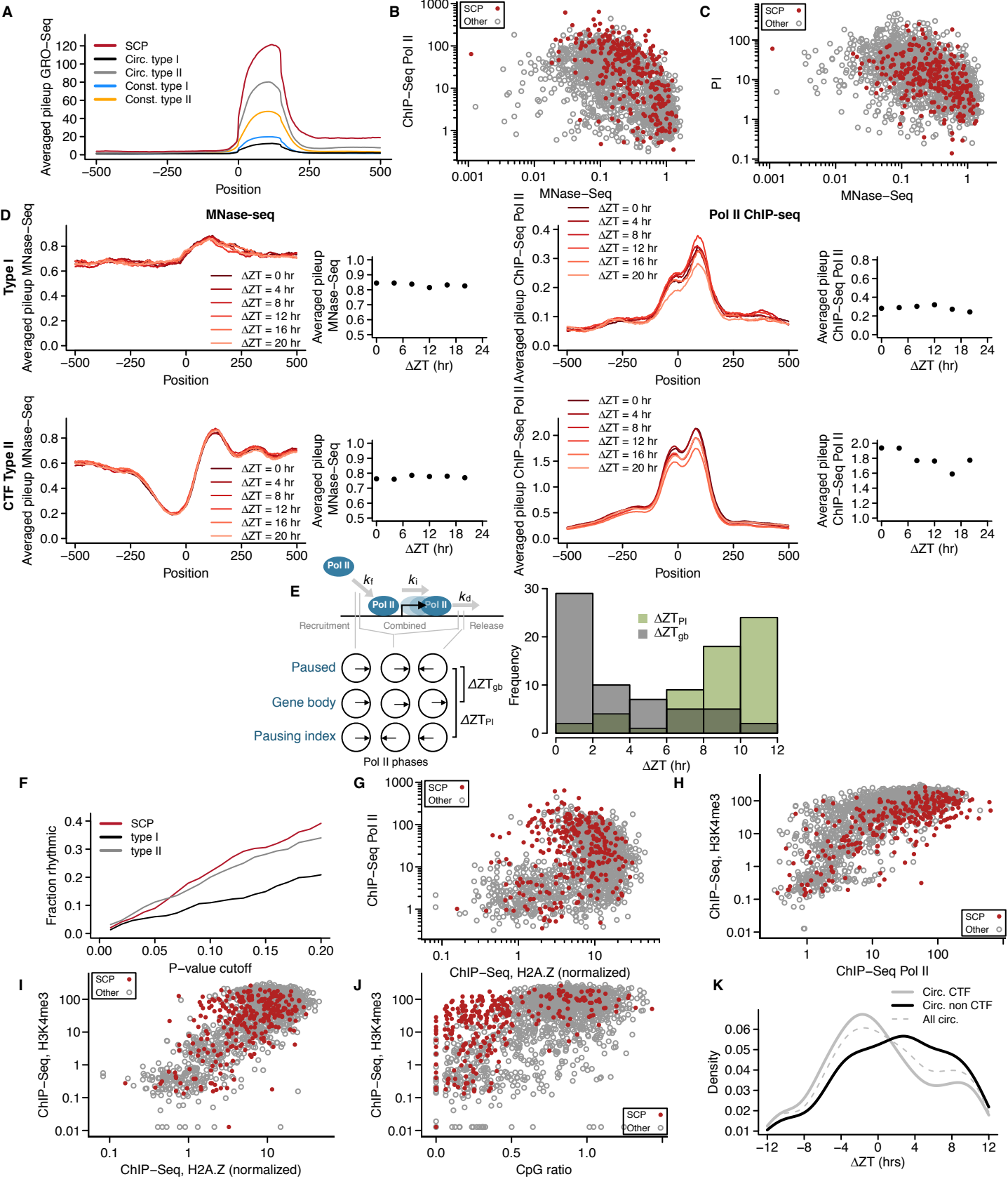

Supplement: S3 Fig — Note that the RPKM Pol II and H3K4me3 values given in the scatter plots here are not equivalent but proportional to the per-base pileup values in Fig 4. See Methods for details. "Other" refers to circadian promoters other than SCPs. A. Averaged pileups of GRO-Seq reads around the TSSs for different promoter classes. Note the "cliff" at around position +150; reads were extended from 50 to 150 bp [6], so that this cliff marks the characteristic pausing position ~50 bp downstream of the TSS. Circ. = Circadian; Const. = Constitutive; Type I = non-CTF binding circadian promoters with TATA box or LCpG, type II = CTF binding circadian promoters without TATA box and with HCpG. B. Levels of Pol II in the promoter-proximal pausing region plotted against averaged nucleosome pileups at between −101 and −1 bp of the TSSs. C. PI plotted against nucleosome pileups as in panel A. D. Oscillations in nucleosome and Pol II pileups as in Fig 4B, but for other promoter groups. E. Phase signatures of circadian Pol II recruitment and release. Left panel: Circles represent phases of paused Pol II, gene body Pol II, and pausing index, respectively. Only a combination of circadian regulation of recruitment and pause release results in paused and gene body Pol II with the same phase, at the same time with paused Pol II and PI with opposite phases (middle column). Not shown is the transition from closed to open DNA-Pol II complex that is also part of the model (S2 Text). Right panel: Estimated phase differences between paused Pol II and gene body Pol II (Δ ZTgb), and between paused Pol II and PI (Δ ZTPI), respectively; details of computation method are given in the Methods section. The observed combination of small Δ ZTgb phase differences and large Δ ZTPI phase differences is only compatible with combined circadian regulation of Pol II recruitment and pause release. F. Fractions of different circadian promoter classes with rhythmic PI. Harmonic regression p values for PI oscillation were comp [file pgen.1006231.s003.pdf]

**A**

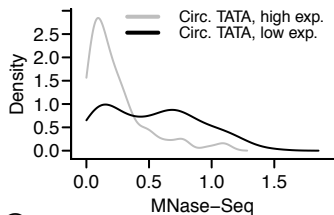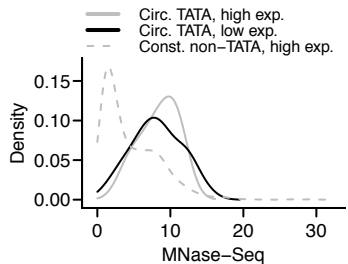

**B**

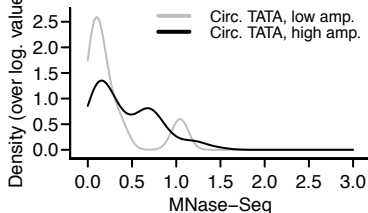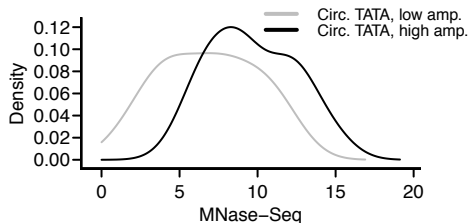

**C**

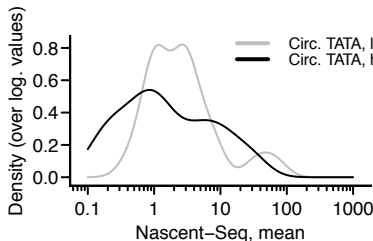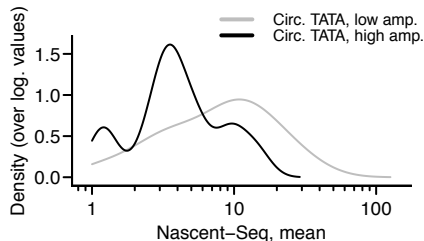

**D**

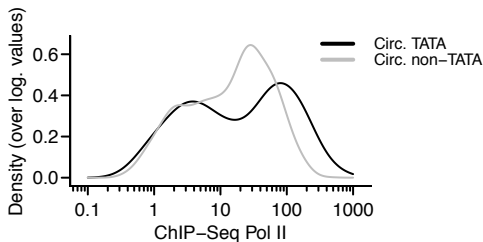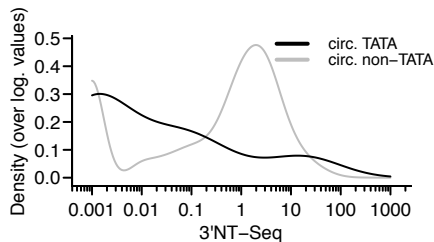

Supplement: S4 Fig — Distributions of nucleosome occupancies, promoter-proximal pol II levels, and transcriptional activities hint at the SCP population in mouse liver, but not in Drosophila. A. Nucleosome occupancies immediately upstream of the TSSs (Methods), stratified for circadian TATA box promoters associated with highly expressed (upper 25% quantile, Methods) transcripts and the other 75%, respectively. All Drosophila circadian promoters, including highly expressed had high nucleosome occupancies, compared to highly expressed constitutive promoters, which had low nucleosome occupancies. On the other hand, in a corresponding plot for mouse liver circadian promoters, the SCP TATA box population is visible with its low nucleosome occupancies. Circ. = Circadian, Const. = Constitutive. B. Comparing nucleosome occupancies immediately upstream of the TSSs for circadian promoters with TATA boxes reveals a subpopulation of mouse promoters with low nucleosome occupancies, but not for Drosophila promoters. Kernel densities of MNase-Seq data (Methods) are visualized. C. For mouse circadian promoters with TATA box, there is a visible subpopulation with high transcriptional activities. Such a subpopulation is not apparent for Drosophila promoters. Kernel densities of Nascent-Seq data (Methods) are visualized. D. For mouse circadian promoters with TATA box, there is a visible subpopulation with high Pol II occupancies in the pausing region immediately downstream of the TSSs (Pol II ChIP-Seq data, Methods). Such a subpopulation is not apparent for Drosophila promoters (3'NT-Seq data, Methods). (PDF) [file pgen.1006231.s004.pdf]

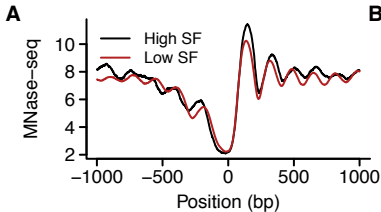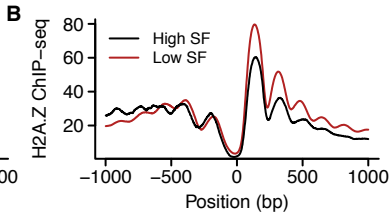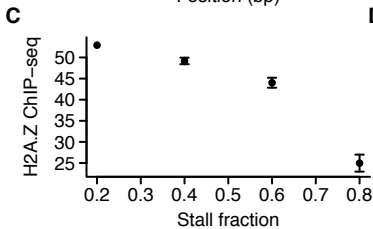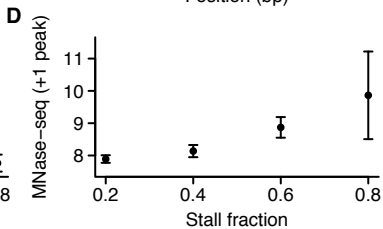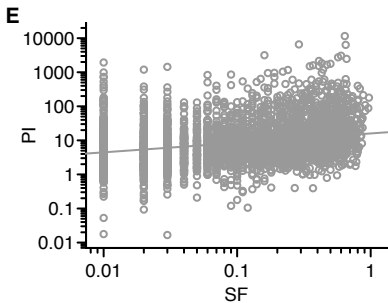

Supplement: S5 Fig — Stall fractions (SFs) measure the percentage of positions with stalled Pol II in the 100 bp upstream of the first nucleosome downstream of the TSS. SFs and nucleosome positions were computed exactly (as far as possible) according to the description in the original article: Stalled positions are defined as 3' end nascent seq peaks rising significantly above their immediate surroundings. The algorithms (SF computation and nucleosome peak detection) were incorporated into the R package "peakPick" (Methods). A. Promoters were classified as having high (SF > 0.6) or low (0.05 < SF ≤ 0.3) SFs. Low SF promoters exhibit slightly lower nucleosome occupancies, which can be interpreted that stalled Pol II is associated with nucleosome barriers. B. Low SF promoters exhibit higher H2A.Z levels (here not normalized to bulk nucleosome levels), which may be interpreted as H2A.Z being associated with less stalling. C and D. Expressed transcripts were binned along SF intervals 0.1–0.3, 0.3–0.5, 0.5–0.7, and 0.7–1. Averaged normalized H2A.Z levels (C) and nucleosome levels (D) at the +1 nucleosome peak are plotted for each bin. Due to outliers, 20% top and bottom values were left out of the averaging for the H2A.Z levels. Error bars refer to SEM. The increased +1 nucleosome levels associated with high SFs must not be confused with the negative correlation between PI and nucleosome occupancies immediately upstream of the TSSs, demonstrated with the same data set as outlined in the main text. E. PI is correlated to SF. For promoters with PI and SF greater than 0, these two measures of Pol II pausing and stalling, respectively, were correlated (Spearman's rho = 0.32, p < 10−15). A standard linear least squares regression line visualizes the correlation. (PDF) [file pgen.1006231.s005.pdf]
