# Supplementary material for: Linking Core Promoter Classes to Circadian Transcription
Source: PLoS Genet. 2016 Aug 9;12(8):e1006231. doi: 10.1371/journal.pgen.1006231 (PMC4978467; doi:10.1371/journal.pgen.1006231)
Supplement: S1 Text — (PDF) [file pgen.1006231.s009.pdf]

S1 Text

A much discussed issue concerns the control over the transcription initiation rate via Pol II recruitment, versus via release of Pol II into productive elongation. The previous study aimed to clarify whether circadian control over transcription involves both these steps [1]. The investigators analyzed the Pol II chip-seq data using a simple but general mathematical model, which describes Pol II recruitment, transcriptional initiation, and release of Pol II into productive elongation (Fig 4C). This analysis seemed to indicate that circadian control over transcription initiation predominantly occurs via Pol II recruitment.

The rigorous analytical and parameter-independent reanalysis of the model performed here verified this (S2 Text and S1 Interactive Text). However, this model analysis also uncovered that a combination of circadian regulation at the Pol II recruitment and release levels would lead to paused and gene body Pol II coverages in phase, as noted earlier [1]. Yet, the PI would oscillate with the opposite phase. Hence, combined circadian control of Pol II recruitment and release could be inferred by detecting this signature (S3E Fig) in the Pol II ChIP-Seq data. In contrast, circadian regulation of only the recruitment stage would lead to weak PI rhythms in phase with the rhythms of paused and gene body Pol II coverages.

The Pol II ChIP-seq data [1] were reanalyzed in order to search for this signature of combined circadian control of Pol II recruitment and release. By estimating phases of paused and gene body-associated Pol II (Methods), the earlier data analysis results [1] could be reproduced: Phases of paused and gene body Pol II were similar (S3E Fig, grey), except for a handful of cases. This indeed suggests that circadian regulation of transcriptional activity exclusively via Pol II release is not common. However, oscillations in the PI was common, especially for CTF binding promoters including SCPs (S3F Fig). Furthermore, the phase difference between paused Pol II and the PI had a strong bias toward ~12 hours for these cases (S3E Fig, green). This is compatible only with the "Combined" scenario (S3E Fig, left).

These results suggest that rhythmic regulation of transcription via rhythmic Pol II release together with rhythmic regulation of Pol II recruitment is a widespread phenomenon. This makes sense: For default parameter combinations of the mathematical model, rhythmic recruitment alone cannot generate higher relative transcriptional amplitudes than 0.5, no matter how strong the recruitment rhythms are. This is not so for all, but for a substantial part of all parameter combinations (S2 Text and S1 Interactive Text), and it is thus conceivable that this is the case for a subset of circadian promoters. Achievement of very high amplitude rhythms in transcriptional activities would in these cases only be possible by additional rhythmic regulation of Pol II release.

References

1. Le Martelot G, Canella D, Symul L, Migliavacca E, Gilardi F, Liechti R, et al. Genome-wide RNA polymerase II profiles and RNA accumulation reveal kinetics of transcription and associated epigenetic changes during diurnal cycles. PLoS Biol. 2012;10: e1001442. doi:10.1371/journal.pbio.1001442
