# Supplementary material for: Linking Core Promoter Classes to Circadian Transcription
Source: PLoS Genet. 2016 Aug 9;12(8):e1006231. doi: 10.1371/journal.pgen.1006231 (PMC4978467; doi:10.1371/journal.pgen.1006231)
Supplement: S2 Text — (PDF) [file pgen.1006231.s010.pdf]

Rhythmic pausing indices: a model analysis.

Pål O. Westermark

Objective

Time-resolved chip-seq experiments for Pol II (Le Martelot et al. *PLoS Biol* **10** 2012) allow quantification of promoter-proximal paused Pol II, as well as transcribing Pol II in gene bodies.

Here, we ask how various kinetic parameters (such as Pol II recruitment or release rates) influence pausing index (PI) and rhythm propagation. Rhythm propagation is assumed to occur between rhythmic Pol II recruitment and transcriptional initiation, or between rhythmic Pol II release from the paused state and transcriptional initiation, or a combination of both.

From the chip-seq measurements, we obtain three readouts: promoter-proximal paused Pol II density, gene body density, and the ratio of the former to the latter (the PI). Since chip-seq experiments were performed at different times of day, we can estimate relative amplitudes and phases for each of the readouts.

Here, we derive phases of these three readouts for **3 rhythm propagation scenarios:** 1. Only oscillating recruitment of Pol II to the promoter. 2. Only rhythmic regulation of release from the paused state. 3. A combination of these two. We do so by analyzing a quantitative model for recruitment of Pol II and transcription initialization from the original publication (Le Martelot et al. *PLoS Biol* **10** 2012). Scenarios 1 and 2 were considered there, where it was shown (as also done more elaborately here below) that scenario 1 will cause promoter-proximal paused Pol II density and gene body density to oscillate with the same phase. Scenario 2 will cause them to oscillate with opposite phases. Since opposite phases were not observed in the chip-seq data, it was concluded that circadian regulation operates at the level of recruitment of Pol II to promoters. Here we show that scenario 3 will lead to in-phase oscillations of promoter-proximal paused Pol II density and gene body density, as observed experimentally and as in scenario 1. We show, however, that scenario 3 will lead to a PI in antiphase to promoter-proximal paused Pol II density and gene body density. Scenario 1 leads to in-phase oscillations in all three readouts. We have found in the experimental data that for a subset of transcripts, the PI is indeed in antiphase to the Pol II densities.

How can the PI be in antiphase to promoter-proximal paused Pol II density and gene body density? Let these two densities have relative amplitudes A_{prom} and A_{gb}, respectively, but the same phase, and form their ratio f(t), which (apart from a constant factor which we here omit) will look like:

$$f[t] = \frac{A_{\text{prom}} \cos\left(\frac{\pi t}{12}\right) + 1}{A_{\text{gb}} \cos\left(\frac{\pi t}{12}\right) + 1}$$

It follows from elementary analysis of the extrema that this ratio will have the same phases as the two densities except if $A_{\text{prom}} < A_{\text{gb}}$, in which case the phases will be opposite. This is illustrated by the plots below. Thus, our objective involves analyzing how the 3 scenarios outlined above lead to different relations between the relative amplitudes of the densities. We will let recruitment of Pol II to the promoter have a relative amplitude A_f. The release from the paused state has a

relative amplitude A_d. We shall analyze how the observables A_{prom} and A_{gb} and the A_{PI}, the relative amplitude of the PI, depend on A_f and A_d. In this way, we can for instance infer from data whether A_d sometimes is greater than zero, which means that Pol II release is regulated in a circadian fashion.

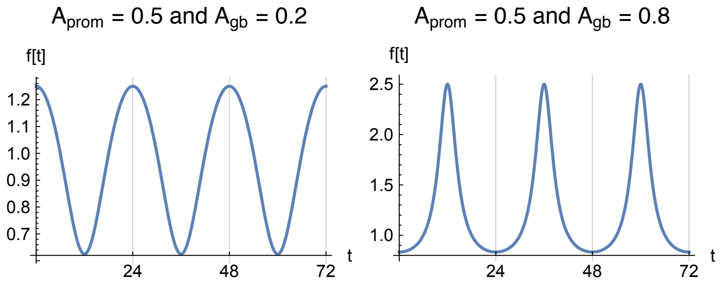

Since we observe such inverted phases of the PI in a subset of transcripts, this analysis allows us to infer that circadian regulation of release from the paused state occurs and is relatively widespread, even if it is not the main cause of circadian rhythms in transcriptional activities.

Finally, we show that rhythm propagation from Pol II recruitment generally weakens when the mean recruitment rate increases. However, when the Pol II initiation rate increases, rhythm propagation increases as well (section **Analysis of transfer functions: effects of parameters on paused Pol II levels and rhythm propagation**). This relates to Figure 4C in the main text.

Model definition and description, parameters

To analyze the consequences of the 3 scenarios, we consider the mathematical model for the initiation of transcription formulated by Le Martelot et al. 2012. This model considers 4 steps: 1, The recruitment of Pol II to chromatin which may be circadian with relative amplitude A_f. 2, Chromatin opening and pre-initiation complex formation of Pol II. 3, Early elongation and formation of promoter-proximal paused Pol II. 4, Release and initiation of transcription from the paused state, which may be circadian with relative amplitude A_d. The model consists of 4 variables, three of them $x + y + z$, represent promoter-associated Pol II, and the fourth, *w,* represents elongating Pol II. The readout $x + y + z$ will exhibit a relative amplitude A_{prom}, and w a relative amplitude A_{gb}.

For a cartoon illustration of the model, see the supplementary material of Le Martelot et al. *PLoS Biol* **10** 2012.

We define this model in *Mathematica* code like so:

```
rhs =
  {kf (1 + u1) (1 - x - y) - kb x - ko x (1 - y), ko x (1 - y) - ki (1 + u3) y (2 - z),
  ki (1 + u3) y (2 - z) - kd (1 + u2) z, kd (1 + u2) z - v w};
vars = {x, y, z, w};
vpars =
  {kf → 1` × 60, kb → 0. × 60, ko → 0.1 × 60 × 60, ki → 1` × 60, kd → 1` × 60, v → 10` × 60};
```

```
Thread[vars == rhs] // MatrixForm
```

$$\begin{pmatrix} x == -kb\,x - ko\,x\,(1-y) + kf\,(1+u1)\,(1-x-y) \\ y == ko\,x\,(1-y) - ki\,(1+u3)\,y\,(2-z) \\ z == ki\,(1+u3)\,y\,(2-z) - kd\,(1+u2)\,z \\ w == -v\,w + kd\,(1+u2)\,z \end{pmatrix}$$

where *u1* represents a relative change in Pol II recruitment rate, and where *u2* represents a relative change in the rate of transcription initiation from the paused state. These two input signals are set to zero when reproducing the original model and computing steady states, but we let them oscillate around zero with amplitudes between 0 and 1 when analyzing the model's reaction to circadian modulation of these rates.

We summarize the model parameters, which we have converted into hours^{-1}:

kf = 60.	kb = 0.	ko = 360.	ki = 60.	kd = 60.	v = 600.

and the differential equations:

$$x'(t) = -\text{kb}\, x + \text{kf}\,(\text{u1}+1)\,(-x-y+1) - \text{ko}\, x\,(1-y)$$
$$y'(t) = \text{ko}\, x\,(1-y) - \text{ki}\,(\text{u3}+1)\, y\,(2-z)$$
$$z'(t) = \text{ki}\,(\text{u3}+1)\, y\,(2-z) - \text{kd}\,(\text{u2}+1)\, z$$
$$w'(t) = \text{kd}\,(\text{u2}+1)\, z - v\, w$$

Initial model code

We are interested in how oscillations in the input signals, that is recruitment (k_f) and release into elongation (k_d), propagate to the variables.

We need the steady state in order to linearize the system for a comprehensive analysis. We solve for the steady state (*u1* = *u2* = 0):

```
vpars =
  {kf → 1` × 60, kb → 0` × 60, ko → 0.1 × 60 × 60, ki → 1` × 60, kd → 1` × 60, v → 10` × 60};
```

```
ss = Flatten[NSolve[Join[
     Thread[rhs == 0] /. vpars /. u1 → 0 /. u2 → 0 /. u3 → 0, Thread[vars > 0]], vars]];
sssym = ss /. Thread[vars → {xss, yss, zss, wss}];
Grid[{sssym}, Frame → True]
```

xss → 0.132677	yss → 0.34943	zss → 0.517893	wss → 0.0517893

We define a state space model object around this steady state (which one can easily linearize and analyze in *Mathematica*) with the 2 inputs *u1* and *u2*, and with 3 outputs: promoter-associated Pol II (*x* + *y* + *z*), transcribing Pol II (*w*), and pausing index (PI): (*x* + *y* + *z*) / *w*.

```
model = NonlinearStateSpaceModel[{rhs, {x + y + z, w, (x + y + z)/w}},
  Thread[vars → {xss, yss, zss, wss}], {u1, u2, u3}]
```

{x, xss}	- kb x - ko x (1 - y) + kf (1 + u1) (1 - x - y)
{y, yss}	ko x (1 - y) - ki (1 + u3) y (2 - z)
{z, zss}	ki (1 + u3) y (2 - z) - kd (1 + u2) z
{w, wss}	- v w + kd (1 + u2) z
	x + y + z
	w
	$\frac{x+y+z}{w}$

We linearize this model like so:

```
modellin = StateSpaceModel[model]
```

■	■	0	0	■	0	0	S
■	■	■	0	0	0	■	
0	■	■	0	0	■	■	
0	0	■	■	0	■	0	
■	■	■	0	0	0	0	
0	0	0	■	0	0	0	
■	■	■	■	0	0	0	

Numerical solution of the model for the 3 scenarios

We start by simulating the 3 scenarios using the model as defined by Le Martelot et al. 2012.

Scenario 1.

Here, we let Pol II recruitment oscillate with relative amplitude $A_f = 0.5$, and let the release into productive elongation have a constant rate ($A_d = 0$). The results are not dependent on parameters or the magnitude of A_f, as outlined further below.

```
Af = 0.5;
Ad = 0`;
vpars = {kf → 60`, kb → 0`, ko → 360`, ki → 60`, kd → 60`, v → 600`};
```

This is the code to solve the model ODEs numerically. We start by computing 100 days to eliminate transients, then compute another 72 hours.

```
simtrans = StateResponse[model /. sssym /. vpars,
    {Af Cos[2 π / 24 * t], Ad Cos[2 π / 24 * t - 0 π / 4]}, {t, 0, 100 * 24}];
simrel = OutputResponse[{model /. sssym /. vpars, simtrans /. t → 72},
    {Af Cos[2 π / 24 * t], Ad Cos[2 π / 24 * t - 0 π / 4]}, {t, 0, 72}];
```

To the three outputs each, a cosine is fitted using the following code, which yields means, relative amplitudes, and phases of the solutions.

```
mycosinor[intfun_] := Module[{fitdata, cosfit, relamp, mean, phase, A, B, M},
    fitdata = {Range[0, 24 - 0.1, 0.1],
        intfun /. t → Range[0, 24 - 0.1, 0.1]}ᵀ;
    cosfit = FindFit[fitdata, A Sin[2 Pi / 24 t] + B Cos[2 Pi / 24 t] + M, {A, B, M}, t];
    relamp = (Sqrt[A^2 + B^2] / M) /. cosfit;
    mean = M /. cosfit;
    phase = ((ArcTan[B, A] /. cosfit) × 24/(2 π)) ~Mod~ 24;
    {mean, relamp, phase}];
```

This function is used to obtain means, amplitudes, and phases for promoter-associated Pol II, gene body Pol II, and PI, respectively:

	Mean	Amp	Phase
Promoter Pol II ($x+y+z$)	0.961605	0.283369	0.0171072
Gene body Pol II (w)	0.0497545	0.238996	0.027955
Pausing index ($(x+y+z)/w$)	19.2157	0.0476102	23.9615

We note that the PI oscillates with very low amplitude in this scenario. We plot the corresponding three outputs:

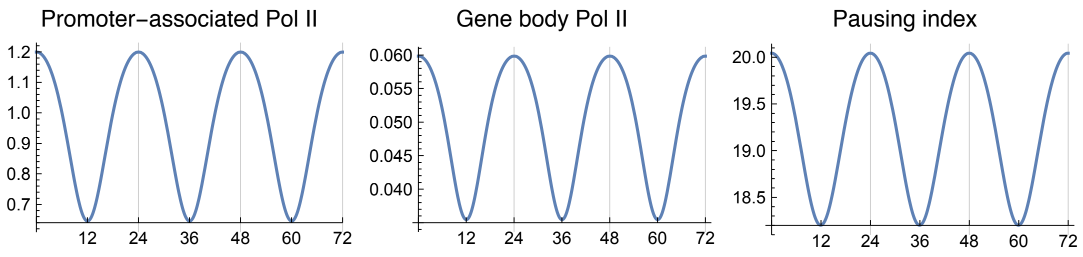

Conclusion: Scenario 1 entails in-phase Pol II and PI. The PI oscillates with such a low amplitude, that it will be hard to detect above noise level experimentally. When analyzing Pol II chip-seq data, we found this scenario to be consistent with observations for most, but not all, circadian genes.

Scenario 2.

Here, we let Pol II recruitment be constant, $A_f = 0$, and let the release into productive elongation oscillate ($A_d = 0.5$). The results are not again not dependent on parameters, as outlined further below.

```
Af = 0`;
Ad = 0.5;
vpars = {kf → 60`, kb → 0`, ko → 360`, ki → 60`, kd → 60`, v → 600`};
```

```
simtrans = StateResponse[model /. sssym /. vpars,
   {Af Cos[2 π / 24 * t], Ad Cos[2 π / 24 * t - 0 π / 4], 0}, {t, 0, 100 * 24}];
simrel = OutputResponse[{model /. sssym /. vpars, simtrans /. t → 72},
   {Af Cos[2 π / 24 * t], Ad Cos[2 π / 24 * t - 0 π / 4]}, {t, 0, 72}];
```

```
Grid[Map[Text[Style[#, FontFamily → "Helvetica"]] &, {
   {"", "Mean", "Amp", "Phase"},
   Flatten@{"Promoter Pol II (x+y+z)", mycosinor[simrel[[1]]]},
   Flatten@{"Gene body Pol II (w)", mycosinor[simrel[[2]]]},
   Flatten@{"Pausing index ((x+y+z)/w)", mycosinor[simrel[[1]] / simrel[[2]]]}
  }, {2}], Frame → True, Alignment → Right]
```

	Mean	Amp	Phase
Promoter Pol II ($x+y+z$)	1.06553	0.282622	12.0192
Gene body Pol II (w)	0.0507848	0.076849	23.8986
Pausing index (($x+y+z$)/w)	21.3034	0.363517	11.9928

We plot the corresponding three outputs:

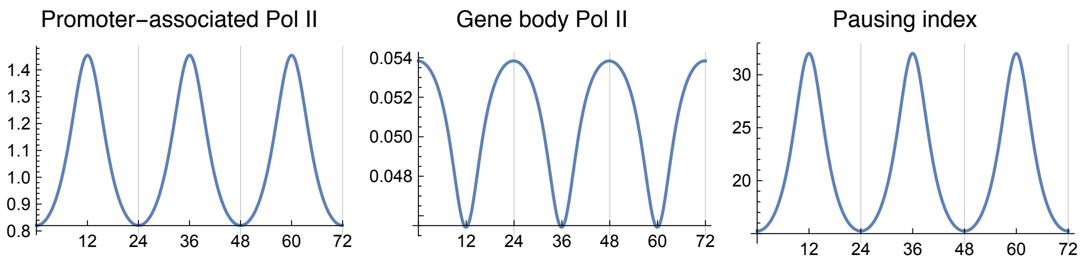

Conclusion: Scenario 2 entails promoter-associated Pol II and gene body Pol II in antiphase. The PI is in phase with promoter-associated Pol II. This is observed for very few genes.

Scenario 3.

Here, we let both Pol II recruitment and release into productive elongation oscillate. The results are not again not dependent on parameters, as outlined further below.

```
Af = 0.6;
Ad = 0.3;
vpars = {kf → 60`, kb → 0`, ko → 360`, ki → 60`, kd → 60`, v → 600`};
```

```
simtrans = StateResponse[model /. sssym /. vpars,
   {Af Cos[2 π / 24 * t], Ad Cos[2 π / 24 * t - 0. π / 4], 0}, {t, 0, 100 * 24}];
simrel = OutputResponse[{model /. sssym /. vpars, simtrans /. t → 72},
   {Af Cos[2 π / 24 * t], Ad Cos[2 π / 24 * t - 0 π / 4]}, {t, 0, 72}];
```

```
Grid[Map[Text[Style[#, FontFamily → "Helvetica"]] &, {
   {"", "Mean", "Amp", "Phase"},
   Flatten@{"Promoter Pol II (x+y+z)", mycosinor[simrel[[1]]]},
   Flatten@{"Gene body Pol II (w)", mycosinor[simrel[[2]]]},
   Flatten@{"Pausing index ((x+y+z)/w)", mycosinor[simrel[[1]] / simrel[[2]]]}
  }, {2}], Frame → True, Alignment → Right]
```

	Mean	Amp	Phase
Promoter Pol II (*x*+*y*+*z*)	0.945376	0.203641	0.0195838
Gene body Pol II (*w*)	0.0493347	0.331337	0.0131052
Pausing index ((*x*+*y*+*z*)/*w*)	19.6221	0.138823	12.0061

We plot the corresponding three outputs:

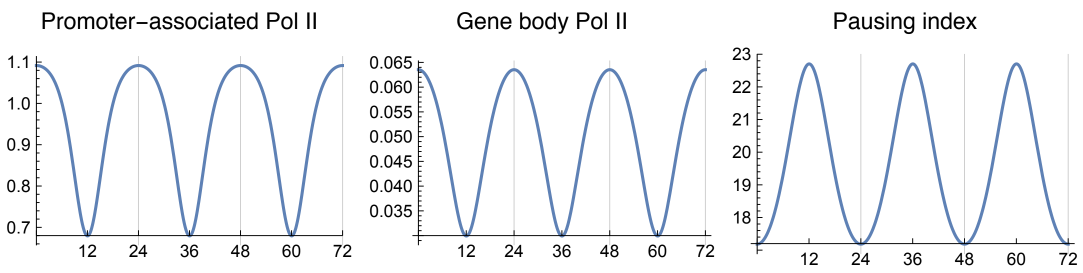

Instead letting Pol II release have the greater amplitude leads to the same results as for scenario 2. In general, starting with scenario 2 and then entering scenario 3 by successively increasing A_f starts leading to the above “inverted PI” phases at a point which is depending on the parameters, as discussed further below.

```
Af = 0.3;
Ad = 0.6;
vpars = {kf → 60`, kb → 0`, ko → 360`, ki → 60`, kd → 60`, v → 600`};
```

```
simtrans = StateResponse[model /. sssym /. vpars,
   {Af Cos[2 π / 24 * t], Ad Cos[2 π / 24 * t - 0 π / 4]}, {t, 0, 100 * 24}];
simrel = OutputResponse[{model /. sssym /. vpars, simtrans /. t → 72},
   {Af Cos[2 π / 24 * t], Ad Cos[2 π / 24 * t - 0 π / 4]}, {t, 0, 72}];
```

```
Grid[Map[Text[Style[#, FontFamily → "Helvetica"]] &, {
   {"", "Mean", "Amp", "Phase"},
   Flatten@{"Promoter Pol II (x+y+z)", mycosinor[simrel[[1]]]},
   Flatten@{"Gene body Pol II (w)", mycosinor[simrel[[2]]]},
   Flatten@{"Pausing index ((x+y+z)/w)", mycosinor[simrel[[1]] / simrel[[2]]]}
  }, {2}], Frame → True, Alignment → Right]
```

	Mean	Amp	Phase
Promoter Pol II (x+y+z)	1.07224	0.204859	12.0228
Gene body Pol II (w)	0.0505833	0.218055	23.971
Pausing index ((x+y+z)/w)	22.3027	0.435745	11.9945

Conclusion: Scenario 3 entails promoter-associated Pol II and gene body Pol II with the same phase. The PI is, however, in antiphase to these Pol II densities. According to the data analyzed, this is widespread although not occurring at the majority of circadian genes.

Transfer function analysis of rhythmic control over transcriptional activity

Are the conclusions above general and parameter-independent? We can derive and analyze the transfer functions from the inputs *u1* (recruitment rate of Pol II) and *u2* (rate of release to productive elongation) to the outputs. This gives us analytic expressions that will tell us, for a given oscillatory input, how variations in kinetic parameters influence levels of paused Pol II (*z*) as well as of rhythm propagation. The expressions will also tell us what phases and amplitudes we can expect in promoter-associated Pol II and elongating Pol II, respectively. These analytical expressions do not rely on fixed parameter values and will indeed show that the conclusions drawn above are general.

We consider relative amplitude gains, which are the ratios of relative amplitudes of the variables to the relative amplitude of oscillation in the input signals. A gain of 0.5 means for the variable x means that if an input signal oscillates with relative amplitude 0.6, then the variable oscillates with relative amplitude 0.6×0.5 = 0.3. Gains can be computed as the absolute values of the transfer functions between the inputs and variables, for the system linearized around the steady states. We derive transfer functions manually using Mason’s rule (Mason SJ (1956) Feedback theory: further properties of signal flow graphs. Proceedings of the IRE 920–926) based on the linearization above, since using *Mathematica*’s built-in TransferFunctionModel function takes too long (symbolic matrix inversion is very slow). **Many of the following details (especially everything between the thin horizontal lines) can be skipped when reading** but are shown in order to follow the process of transfer function derivation with Mason’s rule.

Derivation of transfer functions

First we extract coefficients from the linearization:

```
{A, B} = Normal[modellin][[{1, 2}]];
```

```
Grid[{{("A" == A // TraditionalForm)}, {
   "B" == B // TraditionalForm}}, Frame → True, Alignment → Left]
```

$$A = \begin{pmatrix} -kb - kf - ko\,(1 - yss) & ko\,xss - kf & 0 & 0 \\ ko\,(1 - yss) & -ko\,xss - ki\,(2 - zss) & ki\,yss & 0 \\ 0 & ki\,(2 - zss) & -kd - ki\,yss & 0 \\ 0 & 0 & kd & -v \end{pmatrix}$$

$$B = \begin{pmatrix} kf\,(-xss - yss + 1) & 0 & 0 \\ 0 & 0 & -ki\,yss\,(2 - zss) \\ 0 & -kd\,zss & ki\,yss\,(2 - zss) \\ 0 & kd\,zss & 0 \end{pmatrix}$$

Loops and inverted gains (to simplify the algebra):

```
invgains =
  {iGx → (s - A[[1, 1]]), iGy -> (s - A[[2, 2]]), iGz -> (s - A[[3, 3]]), iGw → (s - A[[4, 4]])};
L1 = A[[2, 1]] A[[1, 2]] Gx Gy;
L2 = A[[3, 2]] A[[2, 3]] Gy Gz;
```

Transfer functions from input *u1* (recruitment rate of Pol II) to the variables. Here, it may look like the loops L1 and L2 decrease the denominators and thus increase the gains, but this is an illusion: this effect is smaller than the negative effect of the loops on the product of the single-variable transfer functions (Gx and so on). These expressions follow from Mason's rule, for which we refer to standard control theory text books or Mason's original paper (Mason SJ (1956) Feedback theory: further properties of signal flow graphs. Proceedings of the IRE 920–926).

```
invrule = {Gx → 1/iGx, Gy → 1/iGy, Gz → 1/iGz, Gw → 1/iGw};
Tx1 = (Gx (1 - L2))/(1 - L1 - L2) /. invrule // FullSimplify;
Ty1 = (Gx A[[2, 1]] Gy)/(1 - L1 - L2) /. invrule // FullSimplify;
Tz1 = (Gx A[[2, 1]] Gy A[[3, 2]] Gz)/(1 - L1 - L2) /. invrule // FullSimplify;
Tw1 = (Gx A[[2, 1]] Gy A[[3, 2]] Gz A[[4, 3]] Gw)/(1 - L1 - L2) /. invrule // FullSimplify;
```

Transfer functions from input *u2* (rate of release to productive elongation) to the variables, separated for action on *z* and on *w*.

```
invrule = {Gx → 1/iGx, Gy → 1/iGy, Gz → 1/iGz, Gw → 1/iGw};
Tx2z = (Gx A[[1, 2]] Gy A[[2, 3]] Gz)/(1 - L1 - L2) /. invrule // FullSimplify;
Ty2z = (Gy A[[2, 3]] Gz)/(1 - L1 - L2) /. invrule // FullSimplify;
Tz2z = (Gz (1 - L1))/(1 - L1 - L2) /. invrule // FullSimplify;
Tw2z = (Gz A[[4, 3]] Gw (1 - L1))/(1 - L1 - L2) /. invrule // FullSimplify;
Tw2w = (Gw (1 - L1 - L2))/(1 - L1 - L2) /. invrule // FullSimplify;
```

Transfer function from input *u3*, the rate of initiation of transcription, before pausing, here we consider only action on *z*.

```
invrule = {Gx → 1 / iGx, Gy → 1 / iGy, Gz → 1 / iGz, Gw → 1 / iGw};
Tz3y = (Gz A[[3, 2]] Gy)/(1 - L1 - L2) /. invrule // FullSimplify;
Tz3z = (Gz (1 - L1))/(1 - L1 - L2) /. invrule // FullSimplify;
```

Analysis of transfer functions: rhythm propagation

Rhythms in Pol II recruitment cause in-phase rhythms in all Pol II densities

We start by looking at all transfer functions from input *u1* – that is, recruitment of Pol II. This section is technical, **the take-home messages are highlighted in bold text**. Material between horizontal lines may be skipped by non-technical readers.

We inspect the transfer functions with expanded expressions for loops L1 and L1, and the inverted single-variable transfer functions (iGx and so on). The latter all have the form $s + \sum k$, where the *k* stands for degradation rate constants.

```
Grid[{{"Tx1" == Tx1 // TraditionalForm},
  {"Ty1" == Ty1 // TraditionalForm}, {"Tz1" == Tz1 // TraditionalForm},
  {"Tw1" == Tw1 // TraditionalForm}}, Frame → True, Alignment → Left]
```

$$\text{Tx1} = \frac{\text{iGy iGz} + \text{ki}^2\,\text{yss}\,(\text{zss}-2)}{\text{iGx}\left(\text{iGy iGz}+\text{ki}^2\,\text{yss}\,(\text{zss}-2)\right)+\text{iGz ko}\,(\text{yss}-1)\,(\text{ko xss}-\text{kf})}$$

$$\text{Ty1} = -\frac{\text{iGz ko}\,(\text{yss}-1)}{\text{iGx}\left(\text{iGy iGz}+\text{ki}^2\,\text{yss}\,(\text{zss}-2)\right)+\text{iGz ko}\,(\text{yss}-1)\,(\text{ko xss}-\text{kf})}$$

$$\text{Tz1} = \frac{\text{ki ko}\,(\text{yss}-1)\,(\text{zss}-2)}{\text{iGx}\left(\text{iGy iGz}+\text{ki}^2\,\text{yss}\,(\text{zss}-2)\right)+\text{iGz ko}\,(\text{yss}-1)\,(\text{ko xss}-\text{kf})}$$

$$\text{Tw1} = \frac{\text{kd ki ko}\,(\text{yss}-1)\,(\text{zss}-2)}{\text{iGw}\left(\text{iGx}\left(\text{iGy iGz}+\text{ki}^2\,\text{yss}\,(\text{zss}-2)\right)+\text{iGz ko}\,(\text{yss}-1)\,(\text{ko xss}-\text{kf})\right)}$$

We note that in particular the factor (ko xss - kf) appears everywhere and is negative in the steady state. **This factor will always be negative:** The first differential equation in the steady state

$$0 = -\text{kb xss} + \text{kf}\,(-\text{xss} - \text{yss} + 1) - \text{ko xss}\,(1 - \text{yss})$$

can be reformulated:

$$0 == (1 - \text{yss})\,(\text{kf} - \text{ko xss}) - \text{kf xss} - \text{kb xss}$$

We see that since 1 – *y* is greater than zero, *(ko xss - kf)* will have to be negative at the steady state.
Since xss and yss are smaller than one, this means that the factor *(ko xss - kf)* always contributes to a decrease of the gains.

We create transfer function objects and normalize by steady-state values. Note that the input already is formulated as relative change. We thus obtain transfer functions between relative amplitudes. We include a transfer function TFp1 for the sum of all promoter-associated Pol II.

```
TFx1 = TransferFunctionModel[B[[1, 1]] Tx1 / xss /. invgains // Simplify, s];
TFy1 = TransferFunctionModel[B[[1, 1]] Ty1 / yss /. invgains // Simplify, s];
TFz1 = TransferFunctionModel[B[[1, 1]] Tz1 / zss /. invgains // Simplify, s];
TFp1 = TransferFunctionModel[
   B[[1, 1]] (Tx1 + Ty1 + Tz1) / (xss + yss + zss) /. invgains // Simplify, s];
TFw1 = TransferFunctionModel[B[[1, 1]] Tw1 / wss /. invgains // Simplify, s];
```

We note that the poles (unit: h^{-1}), which determine dynamics and therefore phase shifts, are far to the left. **This means, phase shifts are small; all variables react with the same phase as the input *u1* (recruitment of Pol II):**

```
TransferFunctionPoles[TFz1 /. sssym /. vpars]
```

```
{{{-271.231, -180.63, -60.}}}
```

The transfer functions for $\omega = 0$ approximate the transfer functions for the circadian frequency $2\pi/24$ h^{-1}, since the poles are some orders of magnitude larger:

```
Grid[{{"Tx1" == TFx1[0] // FullSimplify // TraditionalForm},
  {"Ty1" == TFy1[0] // FullSimplify // TraditionalForm},
  {"Tz1" == TFz1[0] // FullSimplify // TraditionalForm},
  {"Tp1" == TFp1[0] // FullSimplify // TraditionalForm},
  {"Tw1" == TFw1[0] // FullSimplify // TraditionalForm}},
 Frame → True, Alignment → Left]
```

Tx1 =	$\left(\frac{kf\,(-xss-yss+1)\,(ki\,ko\,xss\,yss+kd\,(ko\,xss-ki\,(zss-2)))}{xss\,(ko\,(ko\,xss-kf)\,(yss-1)\,(kd+ki\,yss)+(kb+kf+ko-ko\,yss)\,(ki\,ko\,xss\,yss+kd\,(ko\,xss-ki\,(zss-2))))}\right)$
Ty1 =	$\left(-\frac{kf\,ko\,(-xss-yss+1)\,(yss-1)\,(kd+ki\,yss)}{yss\,(ko\,(ko\,xss-kf)\,(yss-1)\,(kd+ki\,yss)+(kb+kf+ko-ko\,yss)\,(ki\,ko\,xss\,yss+kd\,(ko\,xss-ki\,(zss-2))))}\right)$
Tz1 =	$\left(\frac{kf\,ki\,ko\,(-xss-yss+1)\,(yss-1)\,(zss-2)}{(ko\,(ko\,xss-kf)\,(yss-1)\,(kd+ki\,yss)+(kb+kf+ko-ko\,yss)\,(ki\,ko\,xss\,yss+kd\,(ko\,xss-ki\,(zss-2))))\,zss}\right)$
Tp1 =	$\left(\frac{kf\,(xss+yss-1)\,(kd\,(ko\,(-xss+yss-1)+ki\,(zss-2))+ki\,ko\,(yss^2-(xss+zss-1)\,yss+zss-2))}{(xss+yss+zss)\,(kf\,ki\,ko\,(xss-yss+1)\,yss+kb\,(ki\,ko\,xss\,yss+kd\,(-zss\,ki+2\,ki+ko\,xss))+kd\,(ki\,ko\,(yss-1)\,(zss-2)+kf\,(-zss\,ki+2\,ki+}\right.$
Tw1 =	$\left(\frac{kd\,kf\,ki\,ko\,(-xss-yss+1)\,(yss-1)\,(zss-2)}{v\,wss\,(ko\,(ko\,xss-kf)\,(yss-1)\,(kd+ki\,yss)+(kb+kf+ko-ko\,yss)\,(ki\,ko\,xss\,yss+kd\,(ko\,xss-ki\,(zss-2))))}\right)$

These transfer functions are all positive – this follows from the restrictions on xss, yss, and zss by the model formulation: $x + y < 1$ and $z < 2$. **That the transfer functions are positive means that the phases of all the Pol II variables (promoter- and gene body-associated) are the same as those of the input (recruitment rate)**.

PI has the same phases as all Pol II forms when only Pol II recruitment is rhythmic

What about the PI? At the default parameters, the gain for *w* is smaller than the gain for the sum of the promoter-associated Pol II variables ($x + y + z$):

```
vpars = {kf → 60, kb → 0, ko → 360, ki → 60, kd → 60, v → 600};
```

```
ss = Flatten[NSolve[Join[
      Thread[rhs == 0] /. vpars /. u1 → 0 /. u2 → 0 /. u3 → 0, Thread[vars > 0]], vars]];
sssym = ss /. Thread[vars → {xss, yss, zss, wss}];
Grid[{{"TFp1", TFp1[ⅈ ω] /. vpars /. sssym /. ω → 2 π / 24 // Flatten // First // Abs},
   {"TFw1", TFw1[ⅈ ω] /. vpars /. sssym /. ω → 2 π / 24 // Flatten // First // Abs}}]
```

```
TFp1  0.517888
TFw1  0.425102
```

We note that the magnitudes of the transfer functions fit numerically computed amplitudes above, as they should.

This means that the PI has the same phase as the Pol II densities (see Objective section). This seems to be the case for all parameter combinations: No single counter-example for alternative parameters could be found. To illustrate, we generate 1000 combinations for the 6 model parameters, in log-space, between 1 and 1000 h^{-1}. We do this by using the Sobol algorithm to cover parameter space in an even fashion.

```
parcombs =
  BlockRandom[SeedRandom[Method → {"MKL", Method → {"Sobol", "Dimension" → 6}}];
   RandomReal[{0, 3}, {1000, 6}]];
```

We construct a function that takes one parameter combination and computes the ratios of the absolute values of the transfer functions for the circadian frequency $2\pi/24$ h^{-1}.

```
randomTFratio[sobolexp_] := Module[{vpars, ss, sssym},
    vpars = Thread[{kf, kb, ko, ki, kd, v} → 10^sobolexp];
    ss = Flatten[NSolve[Join[Thread[rhs == 0] /. vpars /. u1 → 0 /. u2 → 0 /. u3 -> 0,
        Thread[vars > 0]], vars]];
    sssym = ss /. Thread[vars → {xss, yss, zss, wss}];
    TFp1val = TFp1[ⅈ ω] /. vpars /. sssym /. ω → 2 π / 24 // Flatten // First // Abs;
    TFw1val = TFw1[ⅈ ω] /. vpars /. sssym /. ω → 2 π / 24 // Flatten // First // Abs;
    TFp1val / TFw1val
   ];
```

We find that all ratios are greater than one, this is the smallest one:

```
Min[randomTFratio /@ parcombs]
```

```
1.00004
```

Thus, relative amplitudes of w are always smaller than for the promoter-associated Pol II variables ($x + y + z$). **And hence, oscillatory input from Pol II recruitment alone will always result in an in-phase PI, according to the curve discussion in the Objective section.**

We justify this observation with an inspection of the analytical formulae:

Keeping in mind that $x + y < 1$ and $z < 2$, the following comparison between the transfer functions for Pol II and the variable z may provide more insight into why the gain for the sum of the promoter-associated Pol II variables ($x + y + z$) is greater. Note that the gain for w is always smaller than that for z, since the step from z to w is linear.

The denominator of the difference of the transfer functions is positive:

```
TFp1[0] - TFz1[0] // Together // Flatten // First // FullSimplify // Denominator
```

$zss\ (xss + yss + zss)\ (kf\ ki\ ko\ (1 + xss - yss)\ yss + kb\ (ki\ ko\ xss\ yss + kd\ (2\ ki + ko\ xss - ki\ zss)) + kd\ (ki\ ko\ (-1 + yss)\ (-2 + zss) + kf\ (2\ ki + ko + ko\ xss - ko\ yss - ki\ zss)))$

So if we can show that the numerator is positive, we have proven the numerically obtained result. Now, in the expression for the numerator below, only the term $ki\,(2\,ko\,(-1 + yss)\,(xss + yss)$ works "against" the expression being positive. However, since we have no closed expressions for the steady-state concentrations (*xss* and so on), a formal mathematical proof cannot be obtained using these methods.

```
num = TFp1[0] - TFz1[0] // Together // Flatten // First // FullSimplify // Numerator
```

$-kf\ (-1 + xss + yss)\ (kd\ ko\ (1 + xss - yss)\ zss + ki\ (2\ ko\ (-1 + yss)\ (xss + yss) + ko\ (xss - 2\ (-1 + yss)\ yss)\ zss - kd\ (-2 + zss)\ zss))$

Rhythmic Pol II release to elongation causes gene body Pol II in phase, but promoter-associated Pol II in antiphase to the release rate

We obtain the **transfer functions from release rate to the different Pol II variables** in a similar way as above:

```
Grid[{{"Tx2z" == Tx2z // TraditionalForm}, {"Ty2z" == Ty2z // TraditionalForm},
  {"Tz2z" == Tz2z // TraditionalForm}, {"Tw2z" == Tw1 // TraditionalForm},
  {"Tw2w" == Tw2w // TraditionalForm}}, Frame → True, Alignment → Left]
```

$$\text{Tx2z} = \frac{ki\ yss\ (ko\ xss - kf)}{iGx\ \left(iGy\ iGz + ki^2\ yss\ (zss - 2)\right) + iGz\ ko\ (yss - 1)\ (ko\ xss - kf)}$$

$$\text{Ty2z} = \frac{iGx\ ki\ yss}{iGx\ \left(iGy\ iGz + ki^2\ yss\ (zss - 2)\right) + iGz\ ko\ (yss - 1)\ (ko\ xss - kf)}$$

$$\text{Tz2z} = \frac{iGx\ iGy + ko\ (yss - 1)\ (ko\ xss - kf)}{iGx\ \left(iGy\ iGz + ki^2\ yss\ (zss - 2)\right) + iGz\ ko\ (yss - 1)\ (ko\ xss - kf)}$$

$$\text{Tw2z} = \frac{kd\ ki\ ko\ (yss - 1)\ (zss - 2)}{iGw\ \left(iGx\ \left(iGy\ iGz + ki^2\ yss\ (zss - 2)\right) + iGz\ ko\ (yss - 1)\ (ko\ xss - kf)\right)}$$

$$\text{Tw2w} = \frac{1}{iGw}$$

These transfer functions tell us which variables increase or decrease, as the release rate is increased, or, for oscillatory input, the phases of the different variables in relation to the phase of oscillation of the release rate.

```
TFx2 = TransferFunctionModel[B⟦3, 2⟧ Tx2z / xss /. invgains // Simplify, s];
TFy2 = TransferFunctionModel[B⟦3, 2⟧ Ty2z / yss /. invgains // Simplify, s];
TFz2 = TransferFunctionModel[B⟦3, 2⟧ Tz2z / zss /. invgains // Simplify, s];
TFp2 = TransferFunctionModel[
    B⟦3, 2⟧ (Tx2z + Ty2z + Tz2z) / (xss + yss + zss) /. invgains // Simplify, s];
TFw2 = TransferFunctionModel[
    (B⟦3, 2⟧ Tw2z + B⟦4, 2⟧ Tw2w) / wss /. invgains // Simplify, s];
```

These transfer functions are:

```
Grid[{{"Tx2" == TFx2[0] // FullSimplify // TraditionalForm},
  {"Ty2" == TFy2[0] // FullSimplify // TraditionalForm},
  {"Tz2" == TFz2[0] // FullSimplify // TraditionalForm},
  {"Tp2" == TFp2[0] // FullSimplify // TraditionalForm},
  {"Tw2" == TFw2[0] // FullSimplify // TraditionalForm}},
 Frame → True, Alignment → Left]
```

$$\text{Tx2} = \left(-\frac{kd\, ki\, (ko\, xss - kf)\, yss\, zss}{xss\, (ko\, (ko\, xss - kf)\, (yss - 1)\, (kd + ki\, yss) + (kb + kf + ko - ko\, yss)\, (ki\, ko\, xss\, yss + kd\, (ko\, xss - ki\, (zss - 2))))} \right)$$

$$\text{Ty2} = \left(-\frac{kd\, ki\, (kb + kf + ko - ko\, yss)\, zss}{ko\, (ko\, xss - kf)\, (yss - 1)\, (kd + ki\, yss) + (kb + kf + ko - ko\, yss)\, (ki\, ko\, xss\, yss + kd\, (ko\, xss - ki\, (zss - 2)))} \right)$$

$$\text{Tz2} = \left(-\frac{kd\, (ko\, (ko\, xss - kf)\, (yss - 1) + (kb + kf + ko - ko\, yss)\, (ko\, xss - ki\, (zss - 2)))}{ko\, (ko\, xss - kf)\, (yss - 1)\, (kd + ki\, yss) + (kb + kf + ko - ko\, yss)\, (ki\, ko\, xss\, yss + kd\, (ko\, xss - ki\, (zss - 2)))} \right)$$

$$\text{Tp2} = \left(-\frac{kd\, zss\, (kb\, (ko\, xss + ki\, (yss - zss + 2)) + kf\, (ko\, (xss - yss + 1) - ki\, (zss - 2)) - ki\, ko\, (yss^2 - (xss + zss - 1)\, yss + zss - 2))}{(ko\, (ko\, xss - kf)\, (yss - 1)\, (kd + ki\, yss) + (kb + kf + ko - ko\, yss)\, (ki\, ko\, xss\, yss + kd\, (ko\, xss - ki\, (zss - 2))))\, (xss + yss + zss)} \right)$$

$$\text{Tw2} = \left(\frac{kd\, ki\, ko\, (kb\, xss + kf\, (xss - yss + 1))\, yss\, zss}{v\, wss\, (ko\, (ko\, xss - kf)\, (yss - 1)\, (kd + ki\, yss) + (kb + kf + ko - ko\, yss)\, (ki\, ko\, xss\, yss + kd\, (ko\, xss - ki\, (zss - 2))))} \right)$$

First, we note that the denominators are all positive, which follows from the fact that $x + y < 1$ and $z < 2$, and that *(ko xss - kf)* is always negative, as discussed above. This allows us to immediately conclude that **the transfer function to the observable gene body Pol II (*w*) is positive (meaning that *w* has the same phase as the release rate)** and that the promoter-associated Pol II variables *y* and *z* are negative (meaning that they have the opposite phase to the release rate). However, the transfer function for *x* is positive, so that this variable will be in phase with the release rate. **The observable *x+y+z* (all promoter-associated Pol II), however, has a negative transfer function and will have a phase opposite to that of the release rate**. This is hinted at by the form of the transfer function but again difficult to prove algebraically. Performing an analysis of 1000 random parameter combinations, generated by the Sobol algorithm as above, justifies this:

```
randomTF2ratio[sobolexp_] := Module[{vpars, ss, sssym},
   vpars = Thread[{kf, kb, ko, ki, kd, v} → 10^sobolexp];
   ss = Flatten[NSolve[Join[Thread[rhs == 0] /. vpars /. u1 → 0 /. u2 → 0 /. u3 -> 0,
       Thread[vars > 0]], vars]];
   sssym = ss /. Thread[vars → {xss, yss, zss, wss}];
   TFp2[i ω] /. vpars /. sssym /. ω → 2 π / 24 // Flatten // First // Arg
  ];
```

Here, since the transfer functions are complex valued, we inspect the absolute values of the arguments (angles) of these numbers, and these are never far from π.

```
Min[Abs[randomTF2ratio /@ parcombs]]
```

```
2.88692
```

Analysis of transfer functions: effects of parameters on paused Pol II levels and rhythm propagation

Increased average Pol II recruitment rate leads to weaker rhythm propagation

We start with the case that only Pol II recruitment is rhythmic, and ask how an increase in the Pol II recruitment rate (k_f), which could explain the higher paused Pol II levels seen in the SCP promoters, affects rhythm propagation. We study what happens for a small (1%) increase of k_f to the gain describing rhythm propagation from recruitment to gene body Pol II.

```
randomTFw1kf[sobolexp_] :=
  Module[{vpars, ss, sssym, vparsmod, ssmod, sssymmod, gaindef, gaindefmod},
   vpars = Thread[{kf, kb, ko, ki, kd, v} → 10^sobolexp];
   ss = Flatten[NSolve[Join[Thread[rhs == 0] /. vpars /. u1 → 0 /. u2 → 0 /. u3 → 0,
       Thread[vars > 0]], vars]];
   sssym = ss /. Thread[vars → {xss, yss, zss, wss}];
   gaindef = (TFw1[i ω]) /. vpars /. sssym /. ω → 2 π / 24 // Flatten // First // Abs;

   vparsmod = vpars /. {(kf → x_) → (kf → x * 1.01)};
   ssmod =
    Flatten[NSolve[Join[Thread[rhs == 0] /. vparsmod /. u1 → 0 /. u2 → 0 /. u3 → 0,
       Thread[vars > 0]], vars]];
   sssymmod = ssmod /. Thread[vars → {xss, yss, zss, wss}];
   gaindefmod =
    (TFw1[i ω]) /. vparsmod /. sssymmod /. ω → 2 π / 24 // Flatten // First // Abs;

   gaindefmod / gaindef
  ];
```

```
allgainratios1kf = randomTFw1kf /@ parcombs;
```

Below we see the fraction of the random parameter combinations have the property that **if the Pol II recruitment rate (**k_f**) is increased, then the rhythm propagation always *decreases*.**

```
Count[allgainratios1kf, x_ /; x < 1] / Length[allgainratios1kf] // N
1.
```

We may also study the combined (summed) rhythm gains from recruitment *and* release to gene body Pol II.

```
randomTFw12kf[sobolexp_] :=
  Module[{vpars, ss, sssym, gaindef, vparsmod, ssmod, sssymmod, gaindefmod},
   vpars = Thread[{kf, kb, ko, ki, kd, v} → 10^sobolexp];
   ss = Flatten[NSolve[Join[Thread[rhs == 0] /. vpars /. u1 → 0 /. u2 → 0 /. u3 → 0,
       Thread[vars > 0]], vars]];
   sssym = ss /. Thread[vars → {xss, yss, zss, wss}];
   gaindef =
    (TFw1[i ω] + TFw2[i ω]) /. vpars /. sssym /. ω → 2 π / 24 // Flatten // First // Abs;

   vparsmod = vpars /. {(kf → x_) → (kf → x * 1.01)};
   ssmod =
    Flatten[NSolve[Join[Thread[rhs == 0] /. vparsmod /. u1 → 0 /. u2 → 0 /. u3 → 0,
       Thread[vars > 0]], vars]];
   sssymmod = ssmod /. Thread[vars → {xss, yss, zss, wss}];
   gaindefmod =
    (TFw1[i ω] + TFw2[i ω]) /. vparsmod /. sssymmod /. ω → 2 π / 24 // Flatten // First //
     Abs;

   gaindefmod / gaindef
  ];
```

```
allgainratios12kf = randomTFw12kf /@ parcombs;
```

Below we see the fraction of the random parameter combinations have the property that if you increase the Pol II recruitment rate (k_f), then the combied rhythm propagation generally *decreases*.

```
Count[allgainratios12kf, x_ /; x < 1] / Length[allgainratios12kf] // N
```

0.867

We see that this is between 85%–90%. This includes the default parameter set (interactive simulation below) and we conclude that **increased Pol II recruitment rates generally weaken rhythm propagation from recruitment *combined with* release.**

Increased average Pol II release rate leads to weaker rhythm propagation from recruitment, may lead to increased propagation from release

Here, we investigate the effect of a small decrease of the Pol II pause release rate on rhythm propagation from recruitment:

```
randomTFw1kd[sobolexp_] :=
  Module[{vpars, ss, sssym, vparsmod, ssmod, sssymmod, gaindef, gaindefmod},
   vpars = Thread[{kf, kb, ko, ki, kd, v} → 10^sobolexp];
   ss = Flatten[NSolve[Join[Thread[rhs == 0] /. vpars /. u1 → 0 /. u2 → 0 /. u3 → 0,
        Thread[vars > 0]], vars]];
   sssym = ss /. Thread[vars → {xss, yss, zss, wss}];
   gaindef = (TFw1[ⅈ ω]) /. vpars /. sssym /. ω → 2 π / 24 // Flatten // First // Abs;

   vparsmod = vpars /. {(kd → x_) → (kd → x * 0.99)};
   ssmod =
    Flatten[NSolve[Join[Thread[rhs == 0] /. vparsmod /. u1 → 0 /. u2 → 0 /. u3 → 0,
        Thread[vars > 0]], vars]];
   sssymmod = ssmod /. Thread[vars → {xss, yss, zss, wss}];
   gaindefmod =
    (TFw1[ⅈ ω]) /. vparsmod /. sssymmod /. ω → 2 π / 24 // Flatten // First // Abs;

   gaindefmod / gaindef
  ];
```

```
allgainratios1kd = randomTFw1kd /@ parcombs;
```

Below we see the fraction of the random parameter combinations have the property that **if the Pol II release rate (k_d) is decreased, then the rhythm propagation always *decreases as well*.**

```
Count[allgainratios1kd, x_ /; x < 1] / Length[allgainratios1kd] // N
```

1.

Then, we investigate the effect of a small decrease of the Pol II pause release rate on rhythm propagation from release only:

```
randomTFw2kd[sobolexp_] :=
  Module[{vpars, ss, sssym, vparsmod, ssmod, sssymmod, gaindef, gaindefmod},
   vpars = Thread[{kf, kb, ko, ki, kd, v} → 10^sobolexp];
   ss = Flatten[NSolve[Join[Thread[rhs == 0] /. vpars /. u1 → 0 /. u2 → 0 /. u3 → 0,
       Thread[vars > 0]], vars]];
   sssym = ss /. Thread[vars → {xss, yss, zss, wss}];
   gaindef = (TFw2[ⅈ ω]) /. vpars /. sssym /. ω → 2 π / 24 // Flatten // First // Abs;

   vparsmod = vpars /. {(kd → x_) → (kd → x * 0.99)};
   ssmod =
    Flatten[NSolve[Join[Thread[rhs == 0] /. vparsmod /. u1 → 0 /. u2 → 0 /. u3 → 0,
       Thread[vars > 0]], vars]];
   sssymmod = ssmod /. Thread[vars → {xss, yss, zss, wss}];
   gaindefmod =
    (TFw2[ⅈ ω]) /. vparsmod /. sssymmod /. ω → 2 π / 24 // Flatten // First // Abs;

   gaindefmod / gaindef
  ];
```

```
allgainratios2kd = randomTFw2kd /@ parcombs;
```

Below we see the fraction of the random parameter combinations have the property that **if the Pol II recruitment rate (k_d) is decreased, then the rhythm propagation from recruitment always increases.**

```
Count[allgainratios2kd, x_ /; x < 1] / Length[allgainratios2kd] // N
```

```
0.
```

Thus the effect of k_d on combined (recruitment + release) rhythmic regulation will be undecided.

Increased average Pol II initiation rate leads to stronger rhythm propagation

Here we ask how an increase in the Pol II initiation rate (k_i) affects rhythm propagation.

```
randomTFw1ki[sobolexp_] :=
  Module[{vpars, ss, sssym, vparsmod, ssmod, sssymmod, gaindef, gaindefmod},
   vpars = Thread[{kf, kb, ko, ki, kd, v} → 10^sobolexp];
   ss = Flatten[NSolve[Join[Thread[rhs == 0] /. vpars /. u1 → 0 /. u2 → 0 /. u3 → 0,
       Thread[vars > 0]], vars]];
   sssym = ss /. Thread[vars → {xss, yss, zss, wss}];
   gaindef = (TFw1[ⅈ ω]) /. vpars /. sssym /. ω → 2 π / 24 // Flatten // First // Abs;

   vparsmod = vpars /. {(ki → x_) → (ki → x * 1.01)};
   ssmod =
    Flatten[NSolve[Join[Thread[rhs == 0] /. vparsmod /. u1 → 0 /. u2 → 0 /. u3 → 0,
       Thread[vars > 0]], vars]];
   sssymmod = ssmod /. Thread[vars → {xss, yss, zss, wss}];
   gaindefmod =
    (TFw1[ⅈ ω]) /. vparsmod /. sssymmod /. ω → 2 π / 24 // Flatten // First // Abs;

   gaindefmod / gaindef
  ];
```

```
allgainratios1ki = randomTFw1ki /@ parcombs;
```

Below we see the fraction of the random parameter combinations have the property that **if the Pol II initiation rate (k_i) is increased, then the rhythm propagation generally *increases*.**

```
Count[allgainratios1ki, x_ /; x > 1] / Length[allgainratios1ki] // N
```

```
0.871
```

We also study the combined (summed) rhythm gains from recruitment *and* release to gene body Pol II.

```
randomTFw12ki[sobolexp_] :=
  Module[{vpars, ss, sssym, gaindef, vparsmod, ssmod, sssymmod, gaindefmod},
   vpars = Thread[{kf, kb, ko, ki, kd, v} → 10^sobolexp];
   ss = Flatten[NSolve[Join[Thread[rhs == 0] /. vpars /. u1 → 0 /. u2 → 0 /. u3 → 0,
       Thread[vars > 0]], vars]];
   sssym = ss /. Thread[vars → {xss, yss, zss, wss}];
   gaindef =
    (TFw1[ⅈ ω] + TFw2[ⅈ ω]) /. vpars /. sssym /. ω → 2 π / 24 // Flatten // First // Abs;

   vparsmod = vpars /. {(ki → x_) → (ki → x * 1.01)};
   ssmod =
    Flatten[NSolve[Join[Thread[rhs == 0] /. vparsmod /. u1 → 0 /. u2 → 0 /. u3 → 0,
       Thread[vars > 0]], vars]];
   sssymmod = ssmod /. Thread[vars → {xss, yss, zss, wss}];
   gaindefmod =
    (TFw1[ⅈ ω] + TFw2[ⅈ ω]) /. vparsmod /. sssymmod /. ω → 2 π / 24 // Flatten // First //
     Abs;

   gaindefmod / gaindef
  ];
```

```
allgainratios12ki = randomTFw12ki /@ parcombs;
```

Below we see the fraction of the random parameter combinations have the property that **if the Pol II initiation rate (k_i) is increased, then the rhythm propagation from recruitment *combined with* release generally *increases*.**

```
Count[allgainratios12ki, x_ /; x > 1] / Length[allgainratios12ki] // N
```

```
0.971
```

Analysis of parameter effects on mean levels (Figure 4C)

Effects of changes in recruitment and release rate constants, respectively, on paused Pol II (*z*) and transcription

We may verify the intuitive results that **increased recruitment rate increases paused Pol II levels. Increased release rate, on the other hand, decreases paused Pol II levels** (recall that (*ko xss* - *kf*) is negative at the steady state as shown above). This follows from the positivity of the following transfer functions:

```
Grid[{{"Tz1" == (TFz1[0] // FullSimplify) // TraditionalForm},
  {"Tz2" == (TFz2[0] // FullSimplify) // TraditionalForm}},
 Frame → True, Alignment → Left]
```

$$\text{Tz1} = \left(\frac{\text{kf ki ko } (-\text{xss}-\text{yss}+1)\ (\text{yss}-1)\ (\text{zss}-2)}{(\text{ko } (\text{ko xss}-\text{kf})\ (\text{yss}-1)\ (\text{kd}+\text{ki yss}) + (\text{kb}+\text{kf}+\text{ko}-\text{ko yss})\ (\text{ki ko xss yss}+\text{kd } (\text{ko xss}-\text{ki } (\text{zss}-2))))\ \text{zss}}\right)$$

$$\text{Tz2} = \left(-\frac{\text{kd } (\text{ko } (\text{ko xss}-\text{kf})\ (\text{yss}-1) + (\text{kb}+\text{kf}+\text{ko}-\text{ko yss})\ (\text{ko xss}-\text{ki } (\text{zss}-2)))}{\text{ko } (\text{ko xss}-\text{kf})\ (\text{yss}-1)\ (\text{kd}+\text{ki yss}) + (\text{kb}+\text{kf}+\text{ko}-\text{ko yss})\ (\text{ki ko xss yss}+\text{kd } (\text{ko xss}-\text{ki } (\text{zss}-2)))}\right)$$

Cf. Figure 4C in the main text.

Increasing the rate of transcriptional initiation increases paused Pol II (z), and thus, transcription initiation rates

Here, the question is how changes in the initiation rate constant *ki* influence the steady state level of *z*.

```
Grid[{{"Tz3y" == Tz3y // TraditionalForm}, {"Tz3z" == Tz3z // TraditionalForm}},
 Frame → True, Alignment → Left]
```

$$\text{Tz3y} = -\frac{\text{iGx ki } (\text{zss}-2)}{\text{iGx } \left(\text{iGy iGz}+\text{ki}^2 \text{ yss } (\text{zss}-2)\right)+\text{iGz ko } (\text{yss}-1)\ (\text{ko xss}-\text{kf})}$$

$$\text{Tz3z} = \frac{\text{iGx iGy}+\text{ko } (\text{yss}-1)\ (\text{ko xss}-\text{kf})}{\text{iGx } \left(\text{iGy iGz}+\text{ki}^2 \text{ yss } (\text{zss}-2)\right)+\text{iGz ko } (\text{yss}-1)\ (\text{ko xss}-\text{kf})}$$

We obtain the transfer functions from release rate to the different Pol II variables in a similar way as above:

```
TFz3 = TransferFunctionModel[
   (B⟦2, 3⟧ Tz3y + B⟦3, 3⟧ Tz3z) / zss /. invgains // Simplify, s];
```

This transfer functions is:

```
Grid[{{"Tz3" == TFz3[0] // FullSimplify // TraditionalForm}},
 Frame → True, Alignment → Left]
```

$$\text{Tz3} = \left(\frac{\text{ki ko } (\text{kb xss}+\text{kf } (\text{xss}-\text{yss}+1))\ \text{yss } (2-\text{zss})}{(\text{ko } (\text{ko xss}-\text{kf})\ (\text{yss}-1)\ (\text{kd}+\text{ki yss}) + (\text{kb}+\text{kf}+\text{ko}-\text{ko yss})\ (\text{ki ko xss yss}+\text{kd } (\text{ko xss}-\text{ki } (\text{zss}-2))))\ \text{zss}}\right)$$

This transfer function is positive, so that **an increase of *ki* always increases *z* and therefore the transcription initiation rate, *kd* × *z*.**

Numerical interactive analysis of transfer functions

Please note that the notebook must be evaluated in *Mathematica* before the interactive panels below display correctly (error messages may be displayed prior to evaluation).

In particular, the user may convince him or herself that the combination of a lowered k_d and v together with increased k_i may explain the combined increased transcriptional activity, higher promoter-proximal Pol II levels, and lower PI levels of SCPs as compared to general Type II promoters (which may be taken as the default parameter set).
Furthermore, the user may convince him or herself that **only** increasing the Pol II recruitment rate, k_f, lowers the net rhythm propagation (Gain sum).

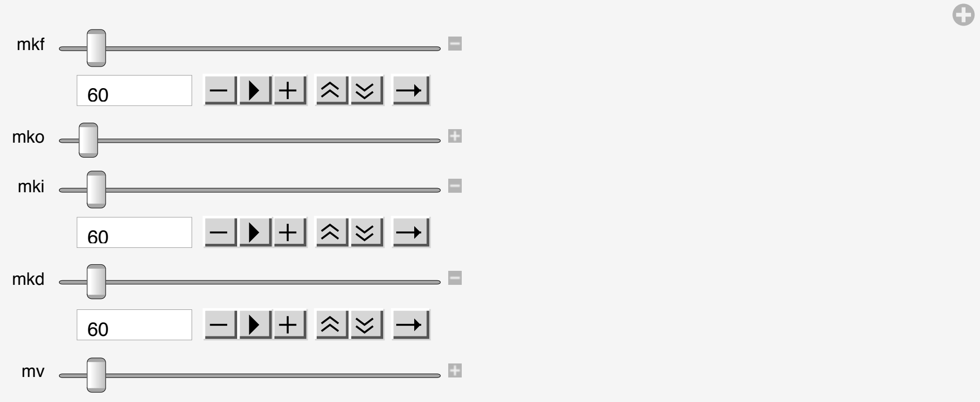

TSS	Promoter	Gene body	PI	Trx init.	Gain recr.	Gain release	Gain sum
0.482107	1.	0.0517893	19.309	31.0736	0.425102	0.120737	0.545774

The following interactive computation allows the reader to manipulate input amplitudes of Pol II recruitment (A_f) and release (A_d), and then directly observe amplitudes and phases of promoter Pol II, gene body Pol II, PI, and transcriptional activity.

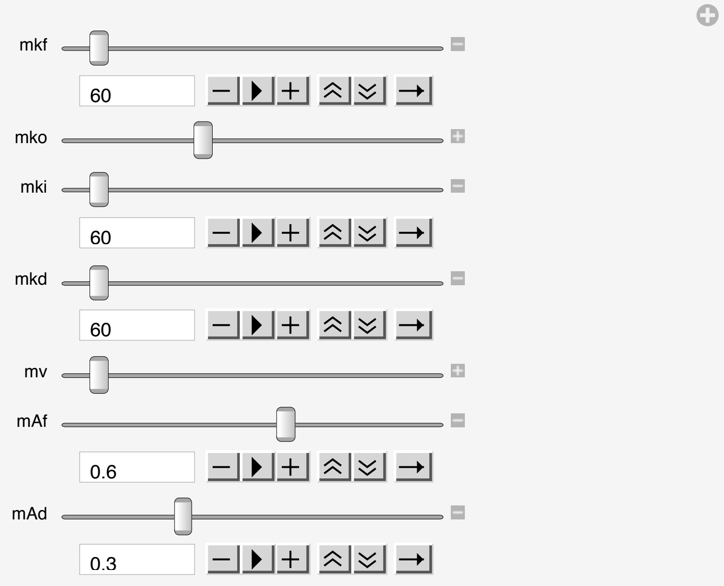

	Mean	Amp	Phase
Promoter Pol II (x+y+z)	0.945376	0.203641	0.0195838
Gene body Pol II (w)	0.0493347	0.331337	0.0131052
Pausing index ((x+y+z)/w)	19.6221	0.138823	12.0061
Trx activity	29.6008	0.331337	0.0131052

Conclusion from the transfer function rhythmicity analysis

The transfer function analysis provided parameter-independent insights into the effects of rhythmic recruitment of Pol II, as well as the effects of rhythmic release of Pol II into productive elongation.

Rhythmic recruitment of Pol II results in rhythms of the same phase in promoter-associated Pol II, gene body Pol II, and PI. The PI phase is the same, since the rhythms are stronger in promoter-associated Pol II than in gene-body Pol II.

Rhythmic release of Pol II results in rhythms with the same phase in gene body Pol II, but with opposite phase in promoter-associated Pol II.

Combined in-phase rhythms in recruitment and release of Pol II result in the recruitment rhythms amplifying the rhythms in gene body Pol II (w) but weakening the rhythms in promoter-associated Pol II ($x+y+z$). For stronger recruitment rhythms and moderate release rhythms, this will result in all Pol II variables being in phase with the recruitment rate, but the gene body rhythms will be stronger than those of promoter-associated Pol II. This results in a PI in antiphase to Pol II densities, as observed in particular for transcripts with strong circadian promoters, and as shown in the numerical example plots above.

Finally, we note that the recruitment gains for gene body Pol II, i.e. the strength with which rhythms in recruitment rates propagate to transcriptional activities, can be low. For the default parameter values, it is 0.5. This means that transcriptional activities cannot reach a larger relative amplitude than 0.5. This is not what is seen in measurements. Rhythmic regulation of release into elongation may thus be needed to produce very high amplitudes. Indeed, this is what our main analysis suggests for a significant percentage of strongly circadian transcripts.
